# MiR-212-3p functions as a tumor suppressor gene in group 3 medulloblastoma via targeting nuclear factor I/B (NFIB)

**DOI:** 10.1186/s40478-021-01299-z

**Published:** 2021-12-18

**Authors:** Naveenkumar Perumal, Ranjana K. Kanchan, David Doss, Noah Bastola, Pranita Atri, Ramakanth Chirravuri-Venkata, Ishwor Thapa, Raghupathy Vengoji, Shailendra K. Maurya, David Klinkebiel, Geoffrey A. Talmon, Mohd W. Nasser, Surinder K. Batra, Sidharth Mahapatra

**Affiliations:** 1 Department of Biochemistry and Molecular Biology, University of Nebraska Medical Center, Omaha, NE 68198 USA; 2 Department of Biomedical Sciences, Creighton University School of Medicine, Omaha, NE 68124 USA; 3 Department of Ophthalmology and Visual Sciences, Washington University School of Medicine, St. Louis, MO 63110 USA; 4 School of Interdisciplinary Informatics, University of Nebraska at Omaha, Omaha, NE 68182 USA; 5 Department of Pathology and Microbiology, University of Nebraska Medical Center, Omaha, NE 68198 USA; 6 Department of Pediatrics, University of Nebraska Medical Center, Omaha, NE 68198 USA

**Keywords:** 17p13.3, c-Myc, Group 3 medulloblastoma, miR-212-3p, Nuclear factor I/B

## Abstract

Haploinsufficiency of chromosome 17p and c-Myc amplification distinguish group 3 medulloblastomas which are associated with early metastasis, rapid recurrence, and swift mortality. Tumor suppressor genes on this locus have not been adequately characterized. We elucidated the role of miR-212-3p in the pathophysiology of group 3 tumors. First, we learned that miR-212-3p undergoes epigenetic silencing by histone modifications in group 3 tumors. Restoring its expression reduced cancer cell proliferation, migration, colony formation, and wound healing in vitro and attenuated tumor burden and improved survival in vivo. MiR-212-3p also triggered c-Myc destabilization and degradation, leading to elevated apoptosis. We then isolated an oncogenic target of miR-212-3p, i.e. NFIB, a nuclear transcription factor implicated in metastasis and recurrence in various cancers. Increased expression of NFIB was confirmed in group 3 tumors and associated with poor survival. NFIB silencing reduced cancer cell proliferation, migration, and invasion. Concurrently, reduced medullosphere formation and stem cell markers (Nanog, Oct4, Sox2, CD133) were noted. These results substantiate the tumor-suppressive role of miR-212-3p in group 3 MB and identify a novel oncogenic target implicated in metastasis and tumor recurrence.

**Supplementary Information:**

The online version contains supplementary material available at 10.1186/s40478-021-01299-z.

## Introduction

Medulloblastoma (MB), the most common malignant brain tumor of childhood, accounts for 20% of pediatric central nervous system (CNS) neoplasms with annual age-adjusted incidence ranging from 0.38 to 0.42 per 100,000 persons [32, 39]. Wingless-type (WNT), sonic hedgehog (SHH), group 3, and group 4 are the classic subgroups of MB with distinctive clinicopathologic and molecular features [37]. The most aggressive tumors fall into group 3 (non-SHH/WNT MB), which account for approximately 25–30% of all MB cases and belong to a high-risk subgroup punctuated by haploinsufficiency of 17p (20–50% incidence), c-Myc amplification (15–20% incidence), and metastases close to or at diagnosis (30–40%), all resulting in very poor prognosis with < 50% 5-year survivorship [3, 25, 38, 45]. Current treatment regimens involve surgical resection followed by a combination of craniospinal radiation and multi-agent chemotherapy, including vincristine, cisplatin, and either cyclophosphamide or lomustine [40, 41]. Recent studies have implicated MB tumor-initiating (stem) cells in evading chemotherapeutic regimens, facilitating recurrence [15, 31]. Recurrence can further reduce 5-year survival to < 10% [25, 43]. Thus, there is an urgent need to understand group 3 MB pathobiology and key signaling pathways involved in disease progression and tumor recurrence to provide a more accurate risk-adapted targeted treatment approach that can mitigate the dismal survivorship of these patients.

Frequent cytogenetic events target the 17p13.3 locus in group 3 MB and are associated with poor prognosis [6, 7, 33]. Along this locus are multiple tumor suppressor genes and microRNAs (miRNAs), short nucleotide noncoding RNAs capable of inhibiting expression of target genes by preventing translation or promoting degradation [2]. We recently reported the tumor-suppressive properties of miR-1253, which lies on the terminal end of this locus, in MB [24]. Similarly along this locus lies miR-212-3p, a tumor suppressor gene in various cancers, including lung cancer [19], hepatocellular carcinoma [5], prostate cancer [56], and glioblastoma [28]. In colorectal cancer [34] and nasopharyngeal carcinoma [20], miR-212-3p targets MnSOD and Sox4, respectively, to prevent metastasis and invasion. In breast cancer, miR-212-3p regulates angiogenesis through Sp1 and VEGFA [27]. It can also sensitize cetuximab-resistant cells to growth inhibition in head and neck squamous cell carcinoma by targeting HBEGF [18]. Other oncogenic targets of miR-212-3p include CTGF [5], Engrailed-2 [56], and SGK3 [28], which are involved in cancer cell proliferation, migration, and invasion.

To date, no studies have examined the role of miR-212-3p in MB pathogenesis. Given its location on a highly afflicted chromosomal locus in high-risk MB, we hypothesized that miR-212-3p possesses tumor-suppressive properties. Here, we have focused on thoroughly elucidating the anti-neoplastic properties of miR-212-3p in group 3 MB and revealed a new oncogenic target of this miR, Nuclear Factor I/B (NFIB).

## Methods

### Human tissue samples and molecular subgrouping of the MB tissues

Frozen and formalin-fixed paraffin-embedded (FFPE) samples of normal cerebellum (pediatric = 12, adult = 5) and pediatric MB specimens (WNT = 1, SHH = 7, group 3 = 10, group 4 = 14, unknown = 7) were collected from the Children’s Hospital and Medical Center and the University of Nebraska Medical Center, Omaha. Tumor samples were sub-classified into four subgroups using genome-wide DNA methylome analysis (Illumina Methylation EPIC 850 K bead arrays) as previously described [24]. Normal cerebellum specimens were obtained at autopsy. For expression profile of HDACs, EZH2, and NFIB, we cross-analyzed our local MB dataset (Kanchan et al*.*, GSE148390) with publicly available MB datasets (Drusco et al*.*, GSE62381; Roth et al*.*, GSE3526; Gilbertson et al*.*, GSE37418; Kool et al*.*, GSE10327; and Weishaupt et al., GSE124814) [9, 24, 52]. For Kaplan–Meier Survival Analysis, we used the R2 database (Cavalli et al*.*, GSE85217) [3].

### Cell lines and cell culture

All cell lines were authenticated using DNA methylation profiling (New York University) and short tandem repeat (STR) DNA profiling (UNMC). MB cell lines, i.e., D283, D341, D425, were maintained in DMEM with 10–20% FBS and 100 µg/mL penicillin/streptomycin; HDMB03 cells were cultured in RPMI with 10% FBS and 100 µg/mL penicillin/streptomycin. Normal Human Astrocytes (Lonza) were maintained using basal medium supplemented with growth factors. Cell lines were maintained in 95% humidity, 37 °C, 5% CO_2_.

### Reagents

MirVana™ miR-212-3p Mimic (Catalog: 4464066; Assay ID: MC10340) and scramble negative control (Catalog: 4464059) were purchased from ThermoFisher Scientific. Silencing RNA for NFIB and EZH2 (Silencer™ Pre-Designed siRNA (Catalog: 4392420); Assay ID: s9495 and s4918, respectively) were obtained from ThermoFisher Scientific. Tet-On-miR-212-3p lentiviral vector and Tet3G (3rd generation) expression lentiviral vector (Vector ID:VB180123-1018bxq) were purchased from Vector Builder Inc. Detailed reagent sections can be found in the Additional file 1.

### MicroRNA quantification

MiR-212-3p expression was quantified using TaqMan™ MicroRNA Reverse Transcription Kit and TaqMan™ Advanced miRNA Assay (Applied Biosystems). Isolated total RNA was reverse transcribed using a specific stem-loop primer for miR-212-3p (Catalog: 4427975, Assay ID: 000515) and RNA U6B (Catalog: 4427975, Assay ID: 001093). After One-step TaqMan RT-PCR, miR-212-3p was quantified and normalized to RNU6B using the delta-delta Ct method.

### DNA methylation profiling

Genome-wide DNA methylome analysis was performed using the Illumina Methylation EPIC 850K bead arrays as previously described [36]. Genomic DNA was extracted from normal cerebellum and MB FFPE samples using RecoverAll™ Total Nucleic Acid Isolation Kit (Invitrogen). Results are presented as percent methylation at each CpG measured.

### De-methylation studies

HDMB03 cells (3 × 10^5^ in 6-well plates) were treated with global de-methylating agent 5-AzaC (5 µM) (5-Aza-2-deoxycytidine; Sigma). Following 96 h of incubation, miR-212-3p expression was analyzed using TaqMan RT-PCR and presented as relative fold expression compared to control.

### Chromatin immunoprecipitation assay

The chromatin immunoprecipitation (ChIP) assay was performed with the Simplechip Enzymatic Chromatin IP kit (CST, Catalog: 9003). Briefly, the digested cross-linked chromatin (10 μg) was subjected to immunoprecipitation with 5 μg of anti-H3K27me3, anti-H3K4me3, anti-H3K9me2, anti-H3K9Ac, or mouse/rabbit IgG control. Purified ChIP DNA was amplified using specific primers (Forward: 5′-GGAGTCCAGCTTCCTCTCCT-3′; and Reverse: 5′-GCTCCTGGGGGTCTTCAC-3′) detecting the CpG-enriched upstream promoter region of human miR-212-3p. Results are presented as relative enrichment normalized to respective input samples.

### Stable inducible miR-212-3p expression system

Stable HDMB03 cells expressing inducible miR-212 with luciferase reporter were generated using Tet-On Inducible lentiviral vector and Tet3G (3rd generation) lentiviral vector (VectorBuilder Inc.). Briefly, lentiviral particles containing Tet3G vector were transduced with group 3 MB cell line (HDMB03 cells). Following hygromycin antibiotic selection (100 µg/ml), stable HDMB03 cells expressing Tet3G were transduced with lentiviral particles containing inducible miR-212 expression system. The pure population of cells expressing inducible miR-212 and luciferase reporter was double-selected using puromycin selection (1 µg/ml) and mCherry positive cell population sorting (flow cytometry).

### MiR-212-3p expression restoration

Group 3 MB cells (3 × 10^5^) were seeded overnight and subsequently serum-starved for 4 h prior to transient transfection using Lipofectamine 2000 (Invitrogen) for 6 h in serum-free media with miR-212-3p mimic (25 nM and 50 nM) or scramble control (25 nM). Following incubation, fresh complete medium was added. For stable induction , HDMB03 cells were treated with or without Doxycycline (Dox, 500 ng/ml) in complete medium and incubated at 37 °C, 5% CO_2_.

### Cell proliferation assay

MTT [3-(4, 5-dimethylthiazol-2-yl)-2, 5-diphenyl- 2H-tetrazolium bromide] and WST-1 [(4-[3-(4-Iodophenyl)-2-(4-nitro-phenyl)-2H-5-tetrazolio]-1,3-benzene sulfonate)] assays were used to determine cell viability in group 3 MB cells. After transfection/Dox treatment, assays were performed at 24–96 h; absorbances were measured using a microplate reader at 570 nm (MTT assay) and 440 nm (WST-1 assay); data were analyzed using the SOFTMAX PRO software (Molecular Devices Corp.).

### Colony formation assay

After transfection/Dox treatment, MB cells (1 × 10^3^ cells/well) were reseeded in 6-well plates and grown for 7–10 days in a humidified atmosphere (95% humidity) at 37 °C and 5% CO_2_. Cells were washed, fixed with 2.5% methanol and stained with 0.5% crystal violet. Cell staining was dissolved using 10% acetic acid and quantified by measuring the absorbance at 590 nm.

### Cell migration and invasion assay

For transwell migration/invasion assay, transfected/Dox treated stable cells (5 × 10^5^ cells) in serum-free media were seeded in the upper chamber of an insert (8 mm pore size; BD Bioscience) coated with Fibronectin (BD Bioscience) or Matrigel (Invasion Chamber Matrigel Matrix) followed by addition of a chemoattractant (10% FBS in complete media) to the lower chamber. After overnight incubation (16 h), cells that migrated/invaded into the lower chamber were stained using Diff-Quik Stain Set (Siemens Healthcare Diagnostics, Inc.); images were captured using an EVOS FL Auto Imaging System (Life Technologies). Results were quantitated by taking an average cell count, measuring cell numbers from four-field/images/well (10 × magnification).

### Wound healing assay

After transient transfection, cells (5 × 10^5^ cells/well) were plated in a 6-well plate, and upon reaching 80% confluence, a vertical scratch was made using a 10 µL pipette tip. For stable cells, 3 × 10^4^ cells/well were seeded in a culture-insert (ibidi culture-insert 2 well), and after overnight incubation the culture-insert was removed and washed with PBS to remove non-adherent cells. Fresh growth medium (with and without Dox (500 ng/ml)) supplemented with 5% serum was added to the plates. The wound closure area was photographed at denoted time intervals using an EVOS FL Auto Imaging System (Life Technologies). Quantitative measurements (% wound closure) were determined by measuring three fields per well.

### Apoptosis and cell cycle analysis

Annexin-V/Cy™5 (BD Biosciences) and propidium iodide (PI) (Roche Diagnostics) were used to measure apoptosis and cell cycle profile as previously described [24]. Briefly, miR-212-3p mimic or scramble control transfected cells were incubated for 96 h. Following incubation, cells were washed and resuspended in calcium-binding buffer consisting of Annexin-V/Cy™5 and PI (apoptosis assay) or fixed with 70% ethanol and stained with PI (cell cycle analysis). Stained cells were analyzed using a FACS Canto™ Flow cytometer (BD Bioscience).

### Western blotting

Protein lysates (30 µg/lane) were separated on 10% SDS-PAGE gels and transferred to PVDF membrane. Following blocking, target proteins were detected by probing overnight at 4°C with antibodies against: NFIB, Bin1 and p19^ARF^ (Abcam); PARP, cleaved PARP, cleaved caspase-3, caspase-3, cyclin D1, CDK4, CDK6, p-Akt-ser473, β-catenin, and CD133 (Cell Signaling Technologies); and total Akt, p-Erk and ERK (Santa Cruz Biotechnology). Then, membranes were washed and incubated with anti-mouse/rabbit IgG secondary antibody (Invitrogen) conjugated with horseradish peroxidase (HRP) at room temperature. Specific proteins were visualized using an enhanced chemiluminescence detection reagent (Pierce; ThermoFisher Scientific, Inc.).

### MiR-212 target prediction

MiRNA target prediction databases, i.e., Targetscan (http://www.targetscan.org) and mirDIP (http://ophid.utoronto.ca/mirDIP/) were used to determine the putative targets of miR-212-3p. Genes that were downregulated upon miR-212-3p restoration in HDMB03 (RNA sequencing analysis) were compared against them. Targets were chosen based on log_2_ fold change < −1 and *p* < 0.05, yielding 37 common gene targets for miR-212-3p.

### Dual-luciferase reporter assay

Primers for 3′UTR-NFIB (Forward: 5′-GCTTGCTAGCTACACACCAGGGT-3′; and Reverse: 5′-TAGCAGTATAGGCTGGATA-3′) were designed using NCBI-Primer-BLAST tool (https://www.ncbi.nlm.nih.gov/tools/primer-blast/) and purchased from Eurofins. Following PCR amplification of NFIB (3′UTR), mutations were created within the seed sequence of NFIB. The resulting 3′UTR-Wild and 3’UTR-Mutant genes were inserted into the XbaI restriction site of pGL3-control vector (Promega) expressing firefly luciferase. Dual-luciferase reporter assay was performed on HDMB03 cells (3 × 10^5^ cells/well) seeded in 12-well plates. Following overnight incubation, cells were co-transfected with 3’UTR-wild-pGL3/3′UTR-Mutant-pGL3 plasmid, pRL-TK plasmid (Promega) expressing Renilla luciferase (internal control), and miR-212-3p mimic/scramble control for 48 h. Luciferase activity was then measured using the Dual-Luciferase Reporter Assay System (Promega) with a Luminometer (Biotek). Results are presented as Luciferase activity (%), where firefly luciferase activity was normalized to Renilla luciferase activity (internal control) for each transfected sample.

### Immunohistochemistry

Unstained tissue slides (3 µm) were deparaffinized in xylene, rehydrated in a series of alcohol dilutions, and heated in citrate buffer (pH 6.2) for 20 min. Following antigen retrieval, slides were incubated with blocking buffer (Horse serum, Vector Labs) for 60 min at room temperature. Subsequently, anti-NFIB (1:200), anti-c-Myc (1:200), anti-Ki-67 (1:200), or anti-Cleaved Caspase 3 (1:200) primary antibody was added and incubated overnight at 4°C. After PBS wash, slides were incubated with secondary antibody (goat anti-rabbit/mouse conjugated with horseradish peroxidase) for 60 min at room temperature. Detection was performed with DAB Peroxidase Substrate Kit (Vector Labs) followed by counterstaining with hematoxylin. Sections were visualized under EVOS FL Auto Imaging System (Life Technologies). Then the tissues were scored according to the intensity of the dye color and the number of positive cells. The method for IHC score was as follows: 0, negative; 1, < 25% positive tumor cells; 2, 25–50% positive tumor cells; 3, 50%-75% positive tumor cells; and 4, > 75% positive tumor cells. Dye intensity was graded as 0 (no color), 1 (light yellow), 2 (light brown), or 3 (brown). Composite scores were derived from product of staining intensity and % positive cells (0–12).

### Sphere formation assay

For primary and secondary spheres, HDMB03 cells (4 × 10^5^ cells/well) were seeded in Ultra-Low Attachment 6-well plates (Corning™ Costar™ Microplates). Cells were maintained in stem cell media (DMEM/F12, 2% B27, 20 ng/mL bFGF, 20 ng/mL EGF, 40 U/ml penicillin, and 40 μg/ml streptomycin), and incubated for 10 days at 37 °C, 5% CO_2_. Following incubation, primary spheres were dissociated into single cell suspension, reseeded, and allowed to grow for another 10 days to form secondary spheres. Spheres having diameter larger than 50 μm were counted.

### Orthotopic medulloblastoma mouse model

All experimental animal procedures were approved by the Institutional Animal Care and Use Committee (IACUC). Briefly, four to six-week-old NSG (NOD-scid Il2rg^null^) mice were anesthetized using ketamine and xylazine (i.p.) and transferred to a stereotactic frame (Stoelting Co, IL, USA). Hamilton syringe (26-gauge needle) with stable Tet-on miR-212 inducible HDMB03 cells (1.0 × 10^5^ cells/5 µl of PBS) was implanted into the cerebellum (2-mm lateral and 2-mm posterior to lambda, and 2.7-mm deep from the surface of the skull), with a speed of 1 µl/min. Needle was removed after 5 min. After surgery, tumor growth, based on total photon flux, was measured using an IVIS Spectrum imaging system (Caliper life sciences; PerkinElmer, MA, USA). Based on tumor size, mice were randomized into two groups (i) control and (ii) DOX treatment group; doxycycline (2 mg daily through oral gavage) induced expression of miR-212-3p. Tumor growth was monitored using an IVIS Spectrum imaging system. On appearance of first neurological symptoms (as per IACUC guidelines), animals were euthanized. Brains were fixed for analysis.

### Statistical analysis

All experiments were conducted in triplicate. Values are presented as mean ± SD. Statistical analyses were performed using Prism 7.0b (GraphPad Software). For data normalization, control group was set at “1” and experimental groups were represented as fold-change compared to control with error bars reflecting deviation from mean triplicate measurements; statistical analyses were conducted prior to normalization. Differences between groups were compared using a two-tailed, unpaired Student’s t-test, Mann–Whitney U test or one-way analysis of variance followed by a least significant difference post hoc test. Statistical significance was established at *p < 0.05, **p < 0.01, and ***p < 0.001.

## Results

### MiR-212-3p expression is epigenetically silenced by histone modifications in group 3 MB

Group 3 tumors belong to a high-risk subgroup punctuated by haploinsufficiency of 17p (20–50% incidence), c-Myc amplification (15–20% incidence), and metastases proximate to diagnosis (30–40%), all resulting in < 50% 5-year survivorship [3, 25, 38, 45]. Our local MB cohort recapitulated these distinguishing findings in group 3 tumors, with a very high frequency of haploinsufficiency of 17p (90%), c-Myc amplification (40%), metastasis proximate to diagnosis (30%), and low 5-year overall survival (40%), and was comparable to a larger MB dataset [3] (Additional file 2: Fig. S1A(i) and (ii)).

**Fig. 1:**
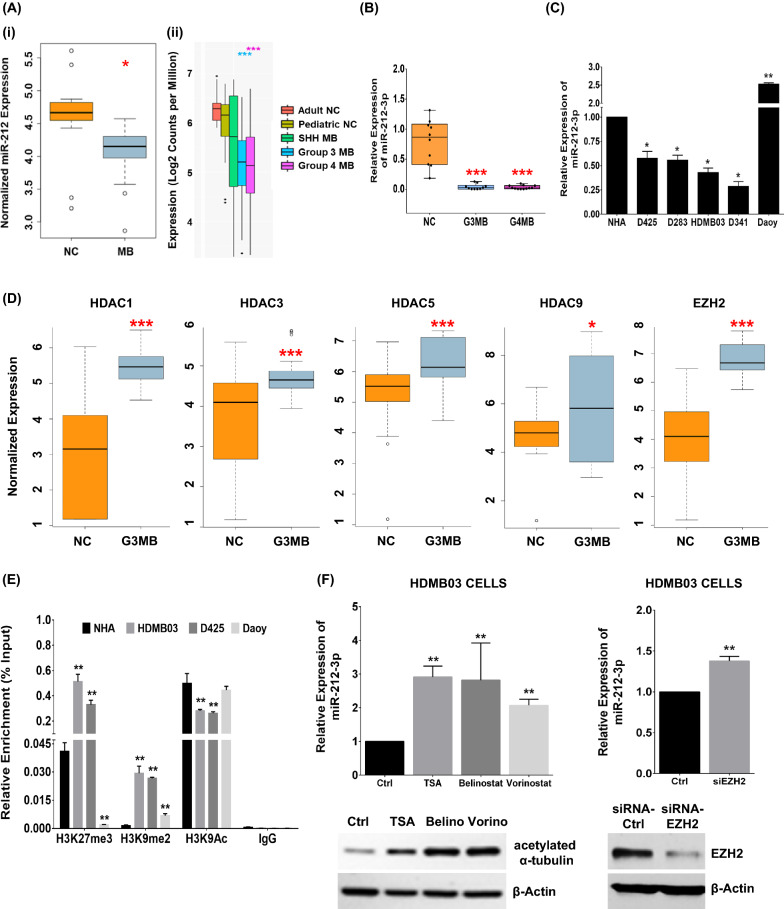
Expression and epigenetic regulation of miR-212-3p in group 3 MB tumors. **A** MiR-212 expression analysis by RNA Sequencing using (i) a publicly available MB dataset (NC n = 14; MB n = 15; Drusco et al*.*, GSE62381) and (ii) a local MB cohort (adult NC n = 4; pediatric NC n = 10; SHH n = 5; group 3 MB n = 9; group 4 MB n = 13; Kanchan et al*.*, GSE148390). **B** Results validated in local MB cohort by RT-PCR (NC n = 10; G3MB n = 10; G4MB n = 12). **C** RT-PCR analysis of miR-212 expression in classic MB cell lines (group 3: D341, D425, HDMB03; group 3/4: D283; SHH: Daoy) compared to normal human astrocytes (NHA). **D** In silico expression of HDACs and EZH2 to investigate epigenetic modulation of miR-212 (NC n = 10; group 3 MB n = 7; Kanchan et al*.*, GSE148390). **E** ChIP-qRT-PCR analysis in group 3 MB cells with deregulated histone modifications in the promoter region of miR-212-3p. ChIP grade histone mark antibodies to H3K27me3, H3K9me2 and H3K9Ac used; IgG antibody as negative control; Daoy cells with high intrinsic miR-212-3p expression served as an additional control. **F** MiR-212-3p expression restoration after treatment with pan-HDAC inhibitors (TSA, 100 nM; Belinostat, 1 µM; and Vorinostat, 1 µM) and with siRNA-EZH2 (20 nM) in HDMB03 cells. Increase in acetylated α-tubulin levels demonstrated pan-HDAC inhibitor activity in HDMB03 cells. β-actin served as an internal control. Data presented as mean ± SD from experiments done in triplicate and analyzed using Mann–Whitney U test (**A** and **D**) or Student’s t-test (**B**, **C**, **E** and **F**); *p < 0.05, **p < 0.01, ***p < 0.001. *NC*, normal cerebellum; *MB*, medulloblastoma; *G3MB*, group 3 medulloblastoma; *G4MB*, group 4 medulloblastoma

To explore the regulation of miR-212-3p in MB, we first conducted an in silico expression analysis of miR-212-3p in CSF samples of patients with MB (NC = 14 vs. MB = 15; GSE62381), revealing reduced expression compared to normal (Fig. 1A (i)). Subsequent expression examination in our local pediatric MB cohort (GSE148390) revealed specific downregulation in group 3 (n = 9) and group 4 (n = 13) MB tumors (Fig. 1A (ii)). We cross validated these results using RT-PCR, noting near-abrogation of expression of miR-212-3p in group 3 MB (n = 10) and group 4 MB samples (n = 12) compared to pediatric cerebellum (n = 10) (Fig. 1B). In vitro, we recapitulated these findings in a panel of MB cell lines with characteristic high-risk features, including i17q and c-Myc amplification (group 3 MB—D341, D425, HDMB03; group 3/4 MB—D283). These were in stark contrast to normal human astrocytes (NHA) and an SHH MB-type cell line, i.e. Daoy, with high expression of miR-212-3p (Fig. 1C). These results substantiated a decreased-to-absent expression of miR-212-3p specific to group 3 and 4 MB tumors and cell lines. We thus chose to focus the rest of our study on group 3 tumors.

To elucidate a mechanism for expression silencing, we studied epigenetic mechanisms, i.e. hypermethylation vs. histone modification. We initially performed DNA methylation profiling in our group 3 MB patient samples (n = 6) but found no perturbation to the methylation of the miR-212-3p promoter region when compared to normal pediatric cerebellum (n = 4). In vitro, these findings were supported by a lack of expression restoration in HDMB03 cells treated with the global demethylating agent, 5-AzaC (5 µM, 96 h) (Additional file 2: Fig. S1B).

Several studies have linked aberrant methylation and acetylation of key histones involved in chromatin structure, including H3K4 and H3K27, with enhanced disease aggressiveness mainly in group 3 and group 4 tumors [10, 22]. Moreover, studies targeting histone de-acetylases (HDACs) have revealed growth inhibition of MYC-driven medulloblastomas [11, 12, 42]. Thus, we shifted our focus to histone modification-mediated epigenetic regulation. Our initial in silico analysis of HDAC expression revealed high expression of HDAC 1, 3, 5, 9, and EZH2 in both our local cohort (group 3 MB n = 7; GSE148390) and in a larger group 3 MB cohort (group 3 MB n = 233; GSE124814) when compared to normal cerebellum (n = 10 and n = 291, respectively) (Fig. 1D and Additional file 2: Fig. S1C). To confirm a specific pattern of histone modification in the predicted miR-212-3p transcription start-site (TSS) in group 3 cells, histone mark patterns of group 3 MB cell lines (HDMB03 and D425) were compared with normal human astrocytes (NHA) and an SHH MB cell line (Daoy). CHIP-qRT-PCR analysis revealed important differences in the methylation status of H3K27 and H3K9 and in the acetylation status of H3K9 in group 3 MB cell lines compared to NHA and Daoy cells (Fig. 1E). Specifically, HDMB03 and D425 cells, with baseline reduced miR-212-3p expression, showed enriched methylated H3K27 and H3K9, with a concomitant decrease in acetylated H3K9, compared to NHA. In contrast, Daoy cells, with high intrinsic miR-212-3p expression, were hypomethylated at H3K27 and showed no change to the acetylation pattern of H3K9 compared to NHA. Consequently, treatment of HDMB03 and D425 cell lines with pan-HDAC inhibitors (TSA, 100 nM; Belinostat, 1 µM; Vorinostat, 1 µM) substantially increased miR-212-3p expression compared to vehicle control (Fig. 1F and Additional file 2: Fig. S1D). Silencing EZH2 expression (siRNA-EZH2, 20 nM) accomplished the same (Fig. 1F). Together, these findings strongly implicated a unique pattern of histone modification as a silencing mechanism for miR-212-3p in group 3 MB tumors.

### MiR-212-3p expression restoration inhibits group 3 MB cell growth and proliferation

**Fig. 2:**
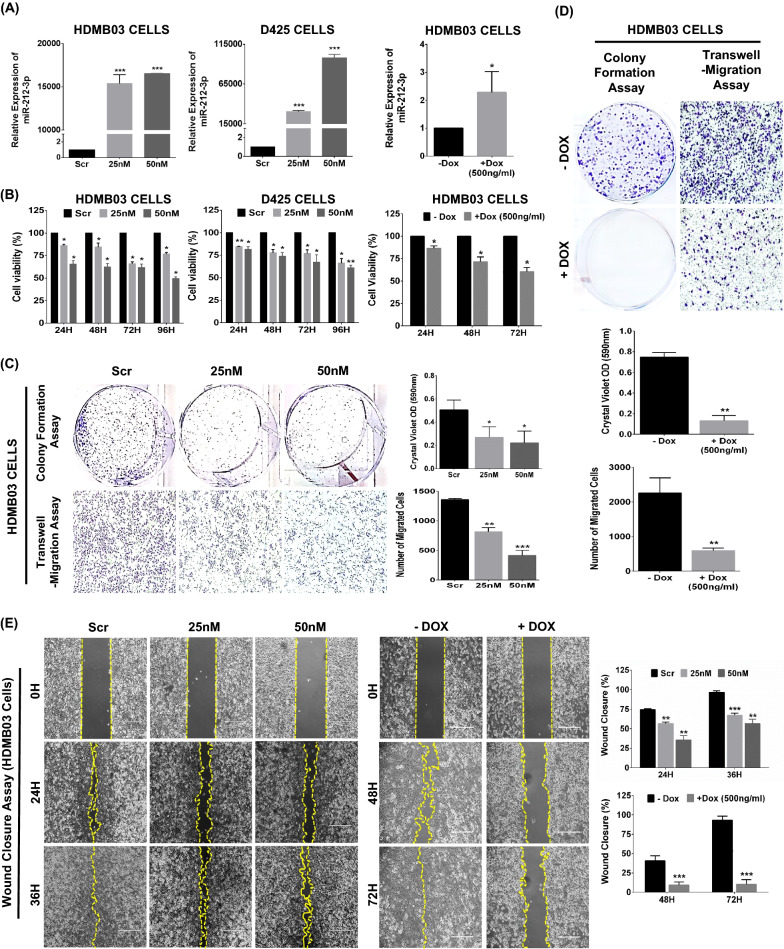
Effect of miR-212-3p expression on the neoplastic potential of group 3 MB cancer cells. **A** MiR-212-3p expression restoration via transient (HDMB03 and D425) and Dox inducible (HDMB03) systems by RT-PCR. **B** Cell proliferation assays (MTT and WST-1) showing significantly reduced proliferative potential with both transient and stable expression of miR-212-3p in a time- and dose-dependent manner in group 3 MB cells. Similar growth-restrictive properties demonstrable via colony formation and transwell migration (**C** and **D**), and wound healing assays (**E**) with both transient and stable expression of miR-212-3p in HDMB03 cells. Data presented as mean ± SD from experiments done in triplicate and analyzed using Student’s t-test; *p < 0.05, **p < 0.01, ***p < 0.001. Scale bar: 100 μm

To highlight the tumor-suppressive properties of miR-212-3p in group 3 MB, we employed two methods to restore miR-212-3p expression, i.e. by transient transfection with miR-212-3p mimic or by Dox-inducible stable expression in group 3 MB cell lines. With successful miR-212-3p expression restoration (Fig. 2A), cancer cell growth and proliferation were significantly impacted in a time- and dose-dependent manner (Fig. 2B). Similarly, colony formation, transwell migration, and wound closure assays all recapitulated this anti-neoplastic phenotype with reduced colonies, cellular migration, and wound closure, respectively (Fig. 2C–E). Although overexpressing miR-212-3p in Daoy cells decreased cell proliferation and colony formation, a dose- and time-dependent effect was absent. Instead, growth inhibitory effects seemed to plateau within 24 h and with a 25 nM concentration of miR-212-3p, suggesting that SHH cell lines do not experience the same growth inhibition as group 3 cell lines do (Additional file 3: Fig. S2A–C). These data provided compelling in vitro evidence for the tumor-suppressive properties of miR-212-3p, expressly in group 3 MB.

### MiR-212-3p induces cell cycle arrest and destabilizes c-Myc to favor apoptosis in group 3 MB

**Fig. 3:**
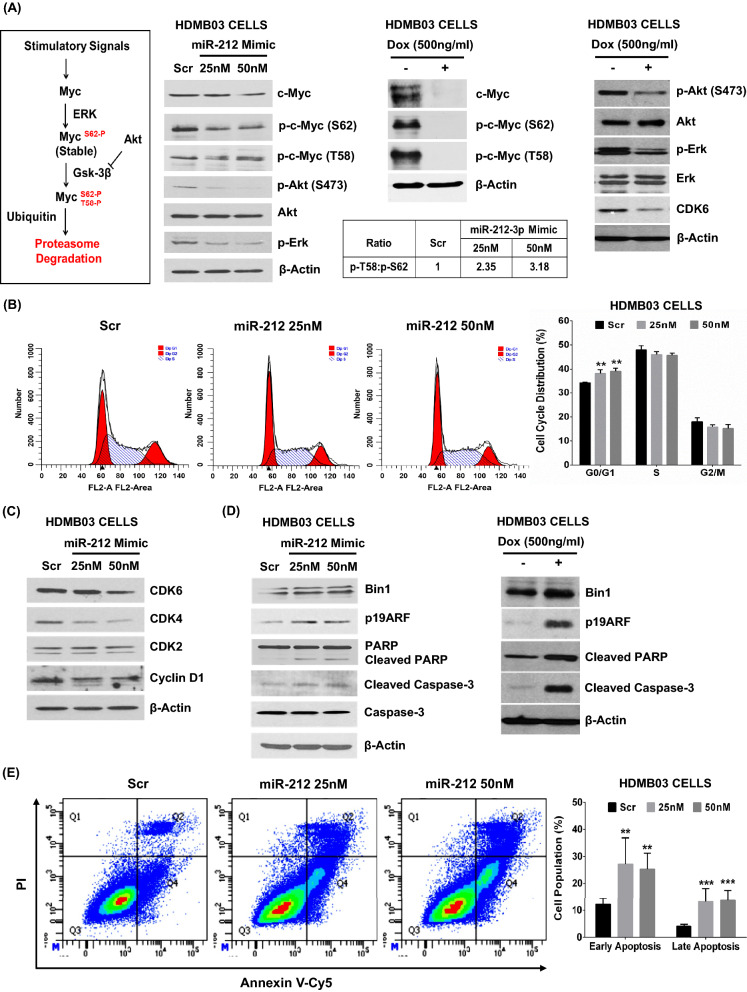
Effect of miR-212-3p expression on c-Myc regulation, cell cycle progression and apoptosis in group 3 MB cancer cells. **A** Western blotting analysis of c-Myc stimulatory signals showing a shift in c-Myc phosphorylation states from serine-62 (active form) to threonine-58 (inactive form) in miR-212-3p transiently transfected HDMB03 cells. In dox-induced stable miR-212 expression, total c-Myc was reduced. Upstream activators of c-Myc, i.e., p-Akt and p-Erk, also destabilized upon miR-212-3p restoration. **B** Cell cycle analysis by propidium iodide (PI) staining showing arrest at G_0_/G_1_ phase in miR-212-3p transiently transfected HDMB03 cells. **C** Western blotting analysis demonstrating reduced expression of G_0_/G_1_ regulatory checkpoint proteins, CDK4, CDK6, and cyclin D1, but not CDK2, in miR-212-3p transiently transfected HDMB03 cells. **D** Elevated expression of pro-apoptotic binding partners of c-Myc, i.e., Bin-1 and p19^ARF^, concurrent with expression of apoptotic proteins (cleaved PARP and cleaved caspase-3) in miR-212-3p restored HDMB03 cells. **E** Annexin-Cy5 and PI staining confirming increased apoptosis (early and late) in miR-212-3p transiently transfected HDMB03 cells compared to scramble control. β-actin served as an internal loading control. Data presented as mean ± SD from experiments done in triplicate and analyzed using Student’s t-test; *p < 0.05, **p < 0.01, ***p < 0.001

Myc amplification is a cardinal high-risk feature of group 3 MB, and its phosphorylation status influences downstream tumor phenotype [43, 44]. More specifically, c-Myc phosphorylated at serine 62 increases stability leading to tumor aggressiveness, while phosphorylation at threonine 58 destabilizes the protein, leading to ubiquitin-mediated degradation and subsequent cellular apoptosis [14, 23]. Transient transfection of HDMB03 cells with miR-212-3p resulted not only in a reduction in total c-Myc protein but also a concomitant increase in the ratio of phosphorylated T58 to phosphorylated S62 (Fig. 3A). Concurrently, the active upstream kinases responsible for c-Myc stabilization via phosphorylation at S62, i.e. p-Erk and p-Akt [46], were both significantly decreased in these cells (Fig. 3A). In miR-212-3p stably-expressing HDMB03 cells, c-Myc expression was almost completely abrogated, with consequent reductions in both phosphorylated species of c-Myc. As prior, p-AKT and p-ERK were also significantly reduced. Together, these data purported a c-Myc deregulatory function for miR-212-3p. Of note, in gastric cancer, c-Myc has been revealed as a direct target of miR-212 [55].

We then focused on progression through the cell cycle, given the destabilization of c-Myc and a reduced proliferative phenotype noted in the presence of miR-212-3p. Not surprisingly, we revealed arrest in transiently-transfected miR-212-3p mimic-treated cells (25 nM and 50 nM) at the G_0_/G_1_ phase of the cell cycle in HDMB03 cells (Fig. 3B). We confirmed the phase of arrest using Western blotting, which showed decreased expression of the complementary checkpoint markers, CDK4, CDK6, and cyclin D1 (Fig. 3A and C). In support, hierarchical clustering and pathways analysis revealed that miR-212-3p expression restoration led to the enrichment of gene clusters (4, 5, 8) involved in the regulation of cell cycle phase (Additional file 4: Fig. S3A and B, and Additional file 5: Table S1).

We concluded our study of the anti-neoplastic properties of miR-212-3p by analyzing its effect on cancer cell apoptosis. We first examined the expression of c-Myc binding partners that signal for apoptosis, i.e. Bin-1 and p19^ARF^, along with various pro-apoptotic proteins. MiR-212-3p restoration in HDMB03 cells significantly increased expression of Bin-1 and p19^ARF^ concurrent with rises in cleaved PARP and cleaved caspase 3 (Fig. 3D). These results were validated using Annexin V-cy5/PI staining, demonstrating a two- and threefold increase in late and early apoptosis, respectively, compared to control (Fig. 3E).

Taken together, our results identified an apoptotic mechanism for miR-212-3p, either by altering c-Myc phosphorylation states to destabilize c-Myc, by reducing the total c-Myc expression, and/or by increasing the expression of its pro-apoptotic binding partners. In parallel, miR-212-3p played a role in cell cycle arrest at the G_0_/G_1_ phase.

### NFIB is a downstream target of miR-212-3p

**Fig. 4:**
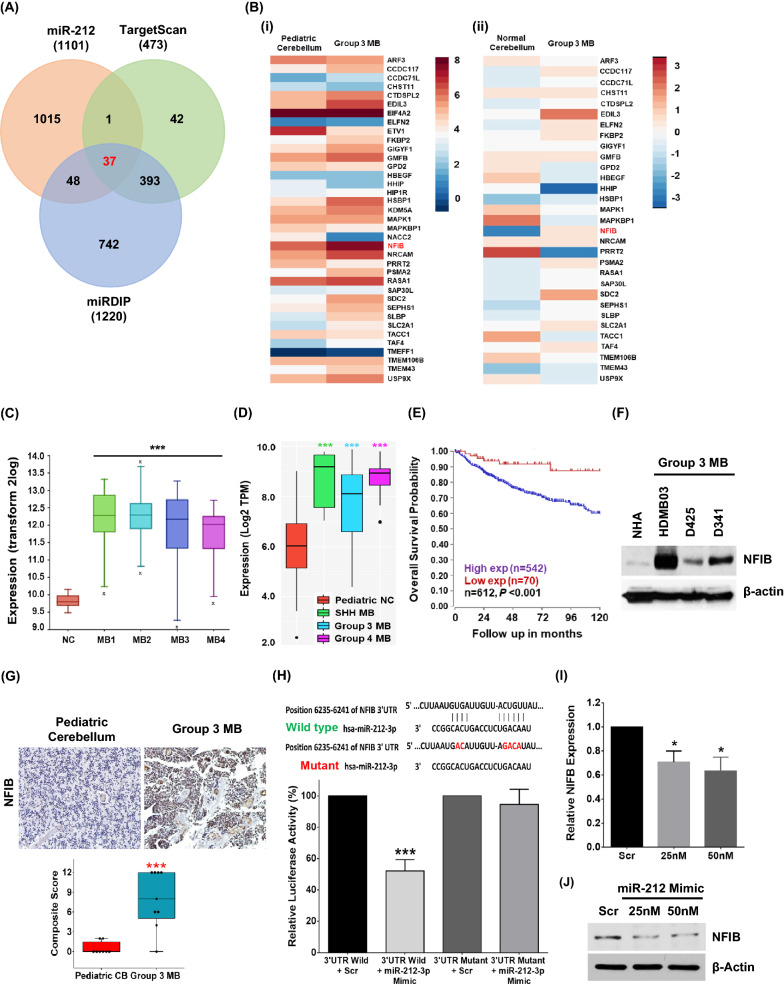
Isolating oncogenic targets of miR-212-3p in group 3 MB tumors. **A** Venn diagram showing 37 common targets between miRNA target prediction databases (Targetscan and miRDip) and genes downregulated (Log_2_ fold change < − 1.0, *p* < 0.05) in miR-212 expressing HDMB03 cells (via RNA sequencing analysis). **B** Expression heat map of the 37 identified targets of miR-212 in two MB data sets, (i) local (pediatric cerebellum n = 10; group 3 MB n = 7; Kanchan et al*.*, GSE148390) and (ii) larger meta-dataset (normal cerebellum n = 291; group 3 MB n = 233; Weishaput et al*.*, GSE124814). **C** Elevated expression of NFIB across MB datasets (NC n = 9, Roth et al. 2008; MB1 n = 76, Gilbertson et al. 2012; MB2 n = 223, Pfister et al. 2017; MB3 n = 57, Delattre et al. 2012; MB4 n = 62, Kool et al. 2009). **D** Elevated NFIB expression across subgroups within local MB cohort (pediatric NC n = 10; SHH n = 6; group 3 n = 7; group 4 n = 12; Kanchan et al*.*, GSE148390). **E** Kaplan–Meier Survival Analysis demonstrating poor survival in high NFIB-expressing MB patients (n = 542) compared to low-expressing patients (n = 70) (Cavalli et al*.*, GSE85217). **F** Western blotting showing significantly increased expression of NFIB in classic group 3 MB cell lines (HDMB03, D425, and D341). **G** Immunohistochemical staining of NFIB showing intense nuclear expression in group 3 MB patient samples (n = 9) when compared to normal cerebellum (n = 8). **H** Dual-luciferase assay confirming NFIB as a direct target of miR-212-3p. **I** RT-PCR and **J** Western blotting analysis showing significantly decreased expression of NFIB in miR-212-3p transiently transfected HDMB03 cells. β-actin served as an internal loading control. Data presented as mean ± SD from experiments done in triplicate and analyzed using Mann–Whitney U test (**C** and **D**) or Student’s t-test (**E**, **G**, and **H**); *p < 0.05, **p < 0.01, ***p < 0.001. Scale bar: 100 μm. *NC*, normal cerebellum

To identify oncogenic targets of miR-212-3p, we started with the targets that are common to miRNA target prediction databases (TargetScan and mirDIP) and that are significantly downregulated (Log_2_ fold change < −1.0, *p* < 0.05) by miR-212-3p expression restoration in HDMB03 cells (Fig. 4A). This comparison revealed 37 putative targets whose expression and associated pathways were studied in two group 3 MB patient cohorts (Fig. 4B (i) and (ii), and Additional file 6: Fig. S4A and B). Out of the 37 common targets, fourteen were upregulated in group 3 MB (summarized in Additional file 7: Table S2). NFIB was chosen for further study based on the following characteristics that bestow a high likelihood of oncogenic potential in group 3 MB: (i) there are two conserved binding sites for miR-212-3p on the 3′UTR of NFIB mRNA (TargetScan); (ii) expression of NFIB was significantly increased across multiple MB datasets (Fig. 4C); (iii) and significantly elevated across MB subgroups (Fig. 4D and Additional file 6: Fig. S4C); (iv) Kaplan–Meier Survival Analysis showed poor survival in high-expressing patients (Cavalli et al*.*, GSE85217, via R2 database) [3] (Fig. 4E); (v) high expression was shown in a panel of group 3 MB cell lines (HDMB03, D425, and D341) compared to NHA (Fig. 4F); and (vi) immunohistochemical staining of NFIB in group 3 MB tissues (n = 9) revealed intense nuclear staining when compared to normal cerebellum (n = 8) (Fig. 4G). We further validated this target by the dual-luciferase assay (Fig. 4H) and showed significant attrition in miR-212-3p mimic treated HDMB03 cells at the transcription (Fig. 4I) and translation levels (Fig. 4J). In this manner, we identified NFIB as an oncogenic target of miR-212-3p.

### NFIB possesses oncogenic potential in group 3 MB cancer cells

Nuclear Factor I/B (NFIB) belongs to the nuclear factor I (NFI) family of transcription and replication proteins that recognize palindromic sequences on various promoters capable of activating transcription and replication throughout organ development [16, 49]. An oncogenic role for NFIB has been shown in triple-negative breast cancer (TNBC), small cell lung cancer (SCLC), colorectal and gastric cancers, and melanoma by enhancing tumor growth, epithelial-mesenchymal transition (EMT), migration, and invasion [13, 29, 30, 47, 53].

**Fig. 5:**
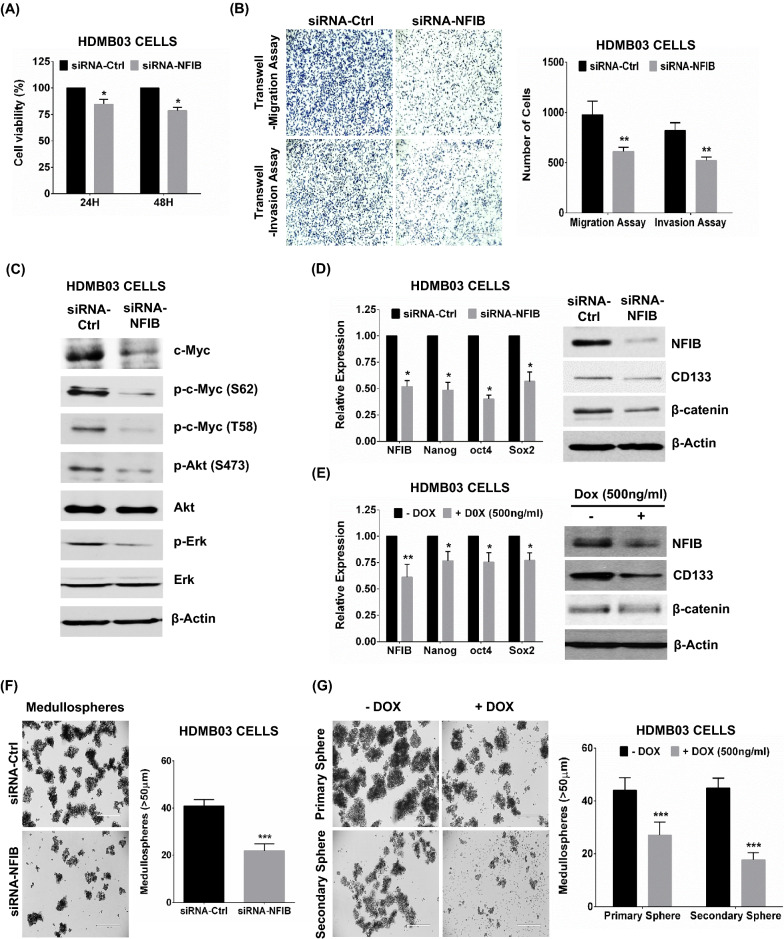
Effect of NFIB silencing on cancer cell phenotype in group 3 MB. **A** Transient silencing of NFIB (siRNA-NFIB, 20 nM) showing significant reduction in HDMB03 cell viability compared to siRNA-control cells. **B** Transwell migration/invasion assay recapitulating the same with decreased migrating and invading cells in siRNA-NFIB-treated cells compared to control. **C** Western blotting analysis of c-Myc showing reduction in total c-Myc, p-S62, p-T58 and reduction in upstream activators of c-Myc, i.e., p-Akt and p-Erk. RT-PCR and Western blotting analyses demonstrating reduced stem cell markers, i.e. Nanog, Oct4, Sox2, CD133 and β-catenin, in **D** siRNA-NFIB-treated HDMB03 cells and **E** Dox-treated stably-expressing miR-212-3p HDMB03 cells. Medullosphere assays demonstrating dramatically reduced sphere formation in **F** siRNA-NFIB-treated and **G** Dox-treated stably-expressing miR-212-3p HDMB03 cells. β-actin served as an internal loading control. Data presented as mean ± SD from experiments done in triplicate and analyzed by Student’s t-test; *p < 0.05; **p < 0.01; ***p < 0.001. Scale bar: 100 μm

With this prior evidence, we elucidated the oncogenic role of NFIB in group 3 MB cancer cells by studying the effect of silencing expression on proliferation, transwellmigration, and invasion. Silencing NFIB in HDMB03 cells transiently resulted in a significant reduction in cell proliferation up to 48 h (Fig. 5A). Transwell migration and invasion assays demonstrated similar reductions in cell migration and invasion (Fig. 5B).

Mechanistically, NFIB overexpression has been shown to increase chromatin accessibility, promote the expression of pro-metastatic genes, and drive metastasis in SCLC tumors [8, 47, 54]. Studies in SCLC have also revealed NFIB as a downstream target of c-Myc, which directly regulates its expression and contributes to tumor aggressiveness and rapid metastases [35]. However, we have shown that miR-212-3p has a deregulatory effect on c-Myc. To delineate if NFIB inhibition plays a role in Myc regulation, we silenced NFIB in group 3 MB cells using transient siRNA-NFIB treatment. As with miR-212-3p stable expression, NFIB silencing decreased total c-Myc levels with a concomitant decrease in phosphorylation of c-Myc at S62 and T58; moreover, upstream c-Myc regulators i.e. p-Akt and p-Erk, were also significantly reduced (Fig. 5C).

Given the direct inhibitory effect of miR-212-3p on c-Myc and NFIB’s promoting role in metastasis, we sought to study the effect of the miR-212-3p/NFIB axis on cancer stem cell (CSC) maintenance and self-renewal. We first analyzed the expression of stem cell markers in miR-212-3p stably-expressing and NFIB-silenced HDMB03 cells. RT-PCR and Western blotting analyses revealed concurrent de-regulation in the expression of several stem cell markers, including CD133, β-catenin, Sox-2, Oct-4, and Nanog (Fig. 5D and E). CSCs self-renewal ability was further assessed through tumor sphere-forming assay. Again, in both NFIB-silenced and miR-212-3p stably-expressing cancer cells, substantially decreased numbers of spheres were noted (Fig. 5F and G). These results further elucidated the anti-neoplastic properties of miR-212-3p with its inhibitory role on CSC maintenance and implicated NFIB as a strong oncogenic target of miR-212-3p in group 3 tumors.

### Restoration of miR-212-3p expression inhibits the growth of orthotopic group 3 MB tumors

**Fig. 6:**
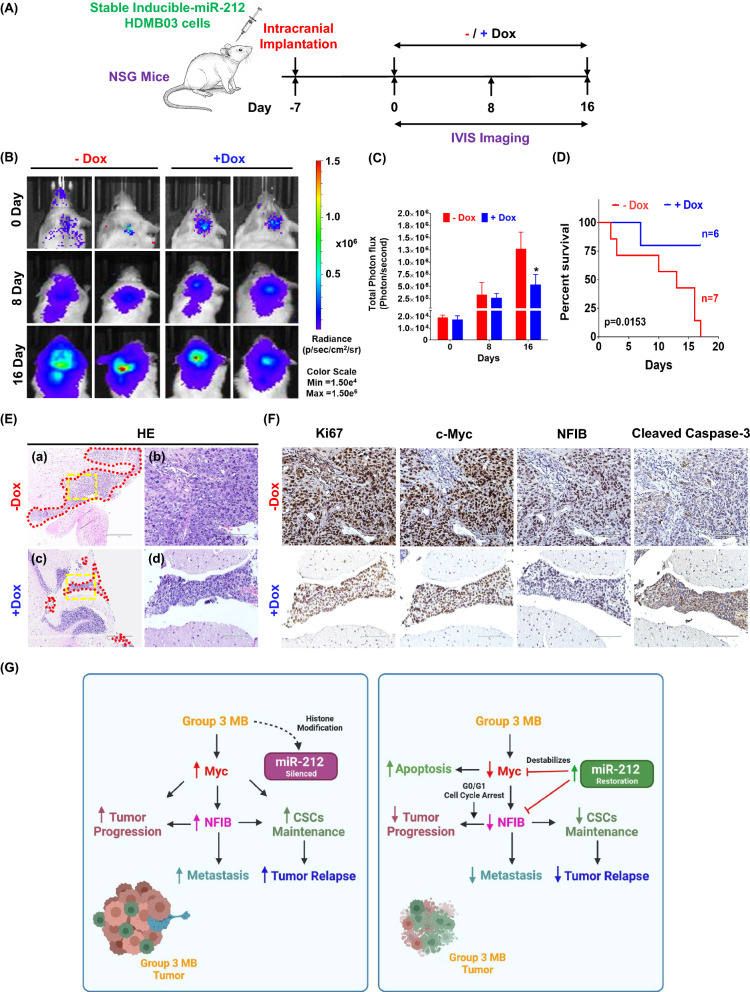
Effect of miR-212-3p expression on in vivo tumorigenicity. **A** Experimental schema depicting generation of orthotopic group 3 MB mouse model with stable Dox induction of miR-212-3p. **B** Bioluminescence images of mice with tumors showing tumor growth on specified days (Day 0, 8 and 16). All images were adjusted to the bioluminescence scale bar shown (Radiance (p/sec/cm^2^/sr); Color scale—Min: 1.50e^4^; Max: 1.50e^6^). **C** Graphical quantification of total photon flux (Photons/second) emitted by tumors on indicated days (Day 0, 8 and 16) between mice with or without Dox treatment, i.e. with or without miR-212-3p expression, respectively. **D** Kaplan-Meier survival analysis of mice with orthotopic tumors showing dramatically attenuated survival in control group (without miR-212-3p induction). **E** Representative histologic images (hematoxylin–eosin stained paraffin sections) of intra-cranial tumors in control and Dox-treated mice. **a** and **c** Area marked (red, 1000 μm) delineates margins of invading tumor. **b** and **d** Magnified images of representative tumor area of **a** and **c** (yellow, 100 µm). **F** Representative images of Ki-67, c-Myc, NFIB and cleaved caspase-3 staining from control and Dox-treated tumors. **G** Schematic representation depicting the tumor suppressive actions of miR-212-3p in group 3 MB. Data presented as mean ± SD from experiments done and analyzed by Student’s t-test; *p < 0.05; **p < 0.01; ***p < 0.001. Kaplan-Meier survival compared between groups by the Log Rank test (*p* = 0.0153). Scale bar: 100 μm

To determine the role of miR-212-3p in group 3 MB tumor growth and progression in vivo, we implanted stable miR-212-inducible HDMB03 cells into the cerebellum of mice as detailed in Fig. 6A. MiR-212-3p expression was restored in mice via daily doxycycline supplementation (oral gavage). At day 16, Dox-fed mice showed significant reduction in tumor growth compared to control (Fig. 6B and C). Moreover, Kaplan-Meier survival analysis revealed significantly higher survival in the Dox-fed group compared to control (Fig. 6D). Tumor histology revealed smaller tumor margins in Dox-fed mice compared to control (Fig. 6E). In addition, Dox-fed mice developed tumors with substantially reduced staining for Ki-67, c-Myc, and NFIB with a concomitant increase in staining for cleaved caspase-3 (Fig. 6F). These in vivo results substantiated the anti-proliferative, pro-apoptotic, and tumor-suppressive properties of miR-212-3p (Fig. 6G) in group 3 MB tumors.

## Discussion

Haploinsufficiency of chromosome 17p bestows a high-risk phenotype upon group 3 tumors [3, 25, 45]. Several loci populated by tumor suppressor genes can be found within the afflicted short arm. Studies have linked few genes residing on the terminal locus (17p13.3) with MB, such as ROX/MNT [7] or HIC1 [51]. None have studied microRNAs on this locus. We previously elucidated the anti-neoplastic properties and oncogenic targets of miR-1253, a microRNA found on the terminal part of this locus, in MB [24]. In the present study, we have described the tumor-suppressive properties of miR-212-3p in group 3 MB; we have additionally identified a target with strong oncogenic potential.

An initial in silico analysis of a publicly available MB dataset revealed significantly reduced expression for miR-212-3p compared to normal. These findings were specifically recapitulated in non-SHH/WNT tumors both in pediatric samples (ex vivo) and in classic MB cell lines (in vitro)*.* Focusing on the higher risk group 3 tumors, we sought a mechanism for miR-212-3p silencing. In many cancers, microRNAs undergo epigenetic silencing via hypermethylation along CpG islands or chromatin rearrangement through histone modifications [24, 26]. Methylation profiling in high-risk tumors showed no differences in methylation pattern of the miR-212-3p locus between tumors and normal tissue. Contrarily, histone modifications at critical lysine residues within miR-212-3p provided compelling evidence for an epigenetic silencing mechanism. More specifically, increased methylation at H3K27 and H3K9 and a concomitant decline in acetylation of H3K9 was shown by ChIP-RT-PCR. Either treatment with HDAC inhibitors or EZH2 silencing reliably restored miR-212-3p expression. Together, these data revealed miR-212-3p silencing in group 3 tumors and assigned an epigenetic mechanism via histone modifications. Our findings aligned with prior reported patterns of miR-212-3p epigenetic silencing in lung cancer [19].

Restoring miR-212-3p expression in group 3 MB cells by either transient transfections or stable induction resulted in a cadre of anti-neoplastic effects. First, cancer cell proliferation, wound healing, migration, and colony formation were significantly reduced. Subsequently, cell proliferative markers, p-Akt and p-Erk, were downregulated in miR-212-3p restored cells, eventually leading to cell cycle arrest (G_0_/G_1_ cell cycle phase) with dysregulated expression of checkpoint regulatory kinases, CDK4, CDK6, and cyclin D1. Next, we revealed regulatory effects of miR-212-3p on c-Myc phosphorylation states, with transient expression resulting in a shift in phosphorylation patterns from S62 (active, favoring proliferation) to T58 (inactive, favoring apoptosis), and stable expression leading to a near-abrogation in total c-Myc expression. Of note, p-Akt and p-Erk, which were downregulated by both transient and stable miR-212-3p expression, are upstream kinases that phosphorylate c-Myc at S62, rendering it resistant to degradation [14, 23]. Moreover, in miR-212-3p-expressing cells, key apoptotic binding partners of c-Myc, p19^ARF^ and Bin-1, were elevated in tandem with cleaved PARP and cleaved caspase‐3, resulting in a high apoptotic signal in cancer cells. As prior, our results aligned with studies on miR-212-3p in colorectal cancer [34], nasopharyngeal carcinoma [20], and lung cancer [21] where similar effects were seen on cancer cell proliferation, migration and invasion, cell cycle arrest, and apoptosis. Given the high association between c-Myc amplification and poor prognosis in group 3 MB [3, 45], our data suggested a plausible mechanism contributing to high-risk MB aggressiveness, wherein silencing miR-212-3p may lift its regulation of c-Myc, allowing for unchecked Myc-driven signaling.

We next identified an oncogenic target of miR-212-3p using in silico, ex vivo*,* and in vitro approaches. In so doing, we revealed Nuclear Factor I/B (NFIB), a transcription factor that can bind to specific overlapping DNA repeat sequences (5′-TTGGCNNNNNGCCAA-3′) throughout the genome to activate transcription and replication, most notably in normal lung and brain development [16, 49]. In fact, the 14 target genes of miR-212 isolated via high-throughput analyses of independent MB datasets seemed most intricately involved in these processes (Additional file 6: Fig. S4B). Given the association of high NFIB expression in group 3 tumors with poor survival, we showed that silencing NFIB expression dramatically decreased cancer cell proliferation, migration, and invasion in vitro. Similar effects have been shown for NFIB in gastric cancers [53], TNBC [29], SCLC [47], and colorectal cancer [30]. In colorectal cancer, melanoma, and gastric cancers, overexpression of NFIB was associated with epithelial-mesenchymal transition (EMT), migration and invasion [13, 30, 53]. In melanoma, specifically, NFIB-targeted upregulation of EZH2 led to the epigenetic silencing of MITF, promoting a highly invasive phenotype [13]. In SCLC, NFIB overexpression cooperated with Rb/p53 deletion to increase chromatin accessibility to pro-metastatic genes. Moreover, c-Myc was shown to regulate NFIB in SCLC and further contribute to rapid metastases [35].

Metastases at diagnosis and tumor recurrence are cardinal prognostic features contributing to dramatically increased mortality in group 3 MB [3, 25, 38, 43–45]. MB recurrent tumors, either in the primary or metastatic site, derive from a subpopulation of cancer stem cells (MBSCs), which can evade current chemotherapeutics and radiation therapy [1]. MBSCs express stemness markers, such as CD133, CD15, and Sox2, and can possess an inordinate capacity to form aggressive tumors with increased self-renewal ability, facilitating MB relapse and rapid demise [48, 50]. In NFIB-silenced and miR-212-3p restored group 3 MB cells, we noted decreased expression of stemness markers, i.e. CD133, Sox2, Oct4, Nanog, and β-catenin, concurrent with reduced tumor cell self-renewal capacity as evidenced by sphere-forming assays. These results introduce miR-212-3p’s role in hampering MBSC maintenance and self-renewal, possibly through NFIB regulation.

These in vitro findings were translated into an orthotopic mouse model wherein miR-212-3p induction led to significantly smaller tumors and substantially elevated survival. Moreover, miR-212-3p-expressing tumors exhibited decreased staining for Ki-67, c-Myc, and NFIB and elevated staining for cleaved caspace-3, strengthening our hypothesis of the tumor-suppressive role of miR-212 in group 3 MB tumors.

Intriguingly, we have shown EZH2 as an upstream regulator of miR-212-3p and revealed miR-212-3p-mediated destabilization of c-Myc. At the core of these critical contributors to tumor aggressiveness lies NFIB, a downstream target of c-Myc (SCLC [35]) and an inducer of EZH2 (melanoma [13]). Whether NFIB plays a role in inducing EZH2 in group 3 tumors to epigenetically silence miR-212-3p, in turn stabilizing c-Myc, is an important mechanism that is being presently evaluated in detail.

## Conclusions

This study has uncovered a novel tumor suppressor gene in group 3 MB, i.e. miR-212-3p. We have shown expression silencing by histone modifications, as opposed to hypermethylation. The anti-neoplastic properties of miR-212-3p were exhibited by destabilization of c-Myc, cancer cell arrest at G_0_/G_1_ cycle, and robust apoptosis, resulting in attrition in cancer cell invasiveness and tumor growth with improved survival in vivo. By targeting NFIB, a well-studied metastatic driver [8], a regulator of EZH2 [13], and downstream effector of c-Myc [35], miR-212-3p decreased cancer cell aggressiveness, stem cell maintenance, and renewal. Our studies provide support for the use of miRNA-based therapies as a targeted approach to not only addressing group 3 MB tumor aggressiveness but also unburdening young patients from the harmful side‐effects of current cytotoxic therapies.

## Supplementary Information

**Additional file 1: Supplemental Methods**

**Additional file 2: Fig. S1.** Exploring epigenetic silencing of miR-212-3p in group 3 MB tumors. (**A**) Frequency distribution of cytogenetic aberrations (haploinsufficiency of 17p, i17q, c-Myc amplification) in (**i**) our local cohort of MB tumors (SHH n=9; group 3 MB n=10; group 4 MB n=16; Kanchan et al., GSE148390). (**ii**) Distinguishing features of high-risk tumors compared between our local cohort (Kanchan et al., GSE148390) and a larger dataset (Cavalli et al., GSE85217). (**B**) DNA methylation profile of local cohort of group 3 (n=6) and group 4 (n=11) MB tumors showing lack of perturbations to methylation in the promoter region of miR-212-3p compared to normal pediatric cerebellum (n=4); further demonstrable in vitro by a lack of expression restoration with de-methylation by 5-AzaC (5 µM) in HDMB03 cells. (**C**) Recapitulation of elevated HDAC and EZH2 expression complementing prior in silico data in a large MB meta-dataset (NC n=291, group 3 MB n=233; Weishaupt et al., GSE124814). (**D**) RT-PCR analysis in pan-HDAC-treated D425 cells (TSA, 100 nM; Belinostat, 1 µM; and Vorinostat, 1 µM) showing elevated expression of miR-212-3p. RNU6B set as an endogenous control. Western blotting analysis showed increased acetylated α-tubulin in pan-HDAC inhibitors treated D425 cells. β-actin served as an internal control. Results presented as mean ± SD from experiments done in triplicate and analyzed using Student’s t-test (**B** and **D**) or Mann-Whiteny U test **(C)**; *p <0.05, **p <0.01, ***p <0.001. *NC*, normal cerebellum; *dx*, diagnosis.

**Additional file 3: Fig. S2.** Effect of miR-212-3p expression on SHH MB cancer cells. (**A**) RT-PCR analysis showing increased miR-212-3p expression via transient transfection in Daoy cells. Growth restrictive properties demonstrable by (**B**) cell proliferation (MTT) and (**C**) colony formation assays in miR-212-3p transfected Daoy cells. Data presented as mean ± SD from experiments done in triplicate and analyzed using Student’s t-test; *p <0.05, **p <0.01, ***p <0.001.

**Additional file 4: Fig. S3.** Deregulated pathways associated with miR-212-3p silencing in group 3 MB. (**A**) Bubble plot (FunSet plot) representing 12 clusters of significantly enriched GO biological processes. FunSet employs hypergeometric test to perform the enrichments and additionally uses semantic similarity measure with Aggregate Information Content (AIC) index to cluster highly similar GO terms [17]. (**B**) Enriched biological pathways associated with miR-212-3p silencing identified using Enrichr (https://maayanlab.cloud/Enrichr/) [4] highlighting regulation of cell cycle phase transition as the most enriched pathway in miR-212-3p overexpressed HDMB03 cells

**Additional file 5: Table S1.** Genes (and associated pathways) enriched by miR-212-3p expression restoration in HDMB03 cells. Detailed gene list for selected clusters of significantly enriched GO biological processes represented in Additional file 4: Fig. S3A. Presented clusters were selected based on -log_10_ FDR values with threshold *p *<0.05.

**Additional file 6: Fig. S4.** Putative oncogenic targets of miR-212-3p and their associated pathways in group 3 MB tumors. (**A**) Expression analysis by RNA Sequencing of isolated miR-212-3p targets (37 genes) in local cohort of group 3 MB tumors (pediatric CB n=10; group 3 MB n=7; Kanchan et al., GSE148390). Those observed to be significantly elevated in group 3 tumors were the following 14 genes: *CCDC71L*, *CTDSPL2*, *EDIL3*, *GIGYF1*, *HSBP1*, *KDM5A*, *NFIB*, *NRCAM*, *PSMA2*, *SDC2*, *SEPHS1*, *TAF4*, *TMEM43*, and *USP9X*. (**B**) Enriched cellular/biological pathways associated with these targets identified using FunSet tool [17]. (**C**) Expression of NFIB across MB subgroups in a large MB meta-dataset (NC n=291; WNT n=118; SHH MB n=405; group 3 MB n=233; group 4 MB, n=530; Weishaupt et al., GSE124814). Box plots (**A**) represent gene expression (Log_2_ Transcripts Per Million) and were analyzed using Mann-Whiteny U test; *p <0.05, **p <0.01, ***p <0.001. Pathways represented as an inverted bar graph based on the -log_10_ FDR values with threshold *p* <0.05 (red dashed line). *CB*, cerebellum; *NC*, normal cerebellum.

**Additional file 7: Table S2.** Isolating a strong oncogenic target of miR-212-3p. Further analysis of putative miR-212-3p targets by examining conserved binding sites, confirming elevated expression in 2 independent MB cohorts, and examining effect of high expression on survival. In the table, ‘x’ denotes inclusion criteria, and ‘-’ denotes no expression data found in dataset.

## Data Availability

Datasets generated by the current study are available from the corresponding author on reasonable request. Datasets used and analyzed during the current study are available in the Gene Expression Omnibus (GEO). These include Kanchan et al*.*, GSE148390; Drusco et al., GSE62381; Roth et al., GSE3526; Gilbertson et al., GSE37418; Kool et al., GSE10327; Weishaupt et al., GSE124814; and Cavalli et al., GSE85217.

## Abbreviations

3′UTR: 3′ Untranslated region
5-AzaC: 5-Aza-2′-deoxycytidine
AKT: Protein kinase B
ANOVA: Analysis of variance
Bin-1: Myc box-dependent-interacting protein 1
c-Myc: Avian myelocytomatosis virus oncogene cellular homolog
CB: Cerebellum
CDK4: Cyclin-dependent kinase 4
CDK6: Cyclin-dependent kinase 6
CNS: Central nervous system
MBSC: Medulloblastoma stem like cells
CTGF: Connective tissue growth factor
DNA: Deoxyribonucleic acid
EMT: Epithelial-mesenchymal transition
EZH2: Enhancer of zeste homolog 2
FACS: Fluorescence-activated cell sorting
FFPE: Formalin-fixed paraffin-embedded
FISH: Fluorescence in situ hybridization
HIC1: Hypermethylated in cancer-1
HCC: Hepatocellular carcinoma
HDAC: Histone de-acetylase
HRP: Horseradish peroxidase
i(17q): Isochromosome 17q
IHC: Immunohistochemistry
IRB: Institutional Review Board
MB: Medulloblastoma
miR: MicroRNA
miR-212-3p: MicroRNA 212-3p
miR-1253: MicroRNA 1253
MITF: Microphthalmia-associated transcription factor
MNT: Max network transcription factor
MTT: 3-(4, 5-Dimethylthiazol-2-yl)-2, 5-diphenyl-2H-tetrazolium bromide
NFIB: Nuclear factor I/B
Nl: Normal
Non-SHH/WNT: Non-sonic hedgehog/non-wingless
NSCLC: Non-small cell lung cancer
Oct4: Octamer-binding transcription factor-4
p19^ARF^: P19 alternative reading frame protein
p-Akt: Phosphorylated protein kinase B
PARP: Poly ADP ribose polymerase
PCR: Polymerase chain reaction
Ped: Pediatric
p-Erk: Phosphorylated extracellular signal related kinase
PI3K: Phosphoinositide 3-kinase
RNA: Ribonucleic acid
SCLC: Non-small cell lung cancer
SHH: Sonic hedgehog
SD: Standard deviation
SGK3: Serum and glucocorticoid-inducible kinase 3
SMYD4: SET and MYND domain containing 4
Sox-2: Sry-box transcription factor-2
TCF7L2: Transcription factor 7-like 2
TNBC: Triple-negative breast cancer
TSA: Tricostatin A
WNT: Wingless
