# Supplementary material for: MiR-212-3p functions as a tumor suppressor gene in group 3 medulloblastoma via targeting nuclear factor I/B (NFIB)
Source: Acta Neuropathol Commun. 2021 Dec 18;9:195. doi: 10.1186/s40478-021-01299-z (PMC8684142; doi:10.1186/s40478-021-01299-z)
Supplement: Supplementary file 1 — Additional file 1: Supplemental Methods [file 40478_2021_1299_MOESM1_ESM.pdf]

Additional File 1: Supplemental Methods

Reagents:

S. No	Antibody name	Manufacturer	Cat. No
1	Cleaved caspase 3	CST	9661
2	Cleaved PARP	CST	9541
3	Ki-67	abcam	ab15580
4	β-actin	Sigma	A1978
5	NFIB	abcam	ab186738
6	NFIB (ChIP Assay)	Sigma	HPA003956
7	H3K27me3 (ChIP Assay)	abcam	ab6002
8	Acetylated α tubulin	CST	5335
9	H3K4me3 (ChIP Assay)	abcam	ab1012
10	H3K9me2 (ChIP Assay)	abcam	ab1220
11	H3K9Ac (ChIP Assay)	abcam	ab32129
12	rabbit IgG control (ChIP Assay)	CST	2729
13	c-Myc	abcam	ab32072
14	Ezh2	CST	5246
15	Anti-c-Myc (Phospho Ser62)	CST	13748
16	Anti-c-Myc (phospho T58)	abcam	ab185655
