# Supplementary figures and images for: MiR-212-3p functions as a tumor suppressor gene in group 3 medulloblastoma via targeting nuclear factor I/B (NFIB)
Source: Acta Neuropathol Commun. 2021 Dec 18;9:195. doi: 10.1186/s40478-021-01299-z (PMC8684142; doi:10.1186/s40478-021-01299-z)

Additional File 2: Fig. S1

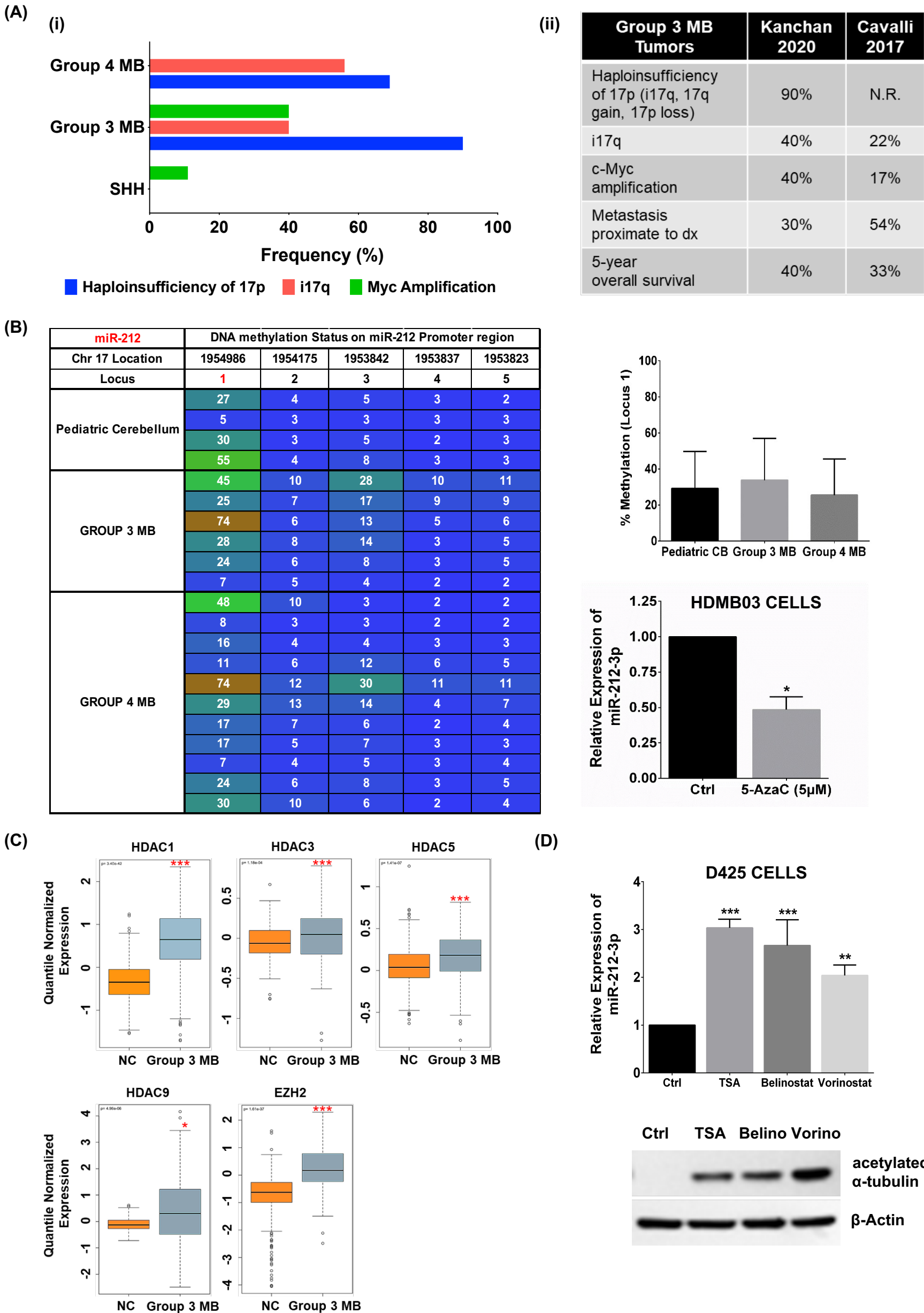

Supplement: Supplementary file 2 — Additional file 2: Fig. S1. Exploring epigenetic silencing of miR-212-3p in group 3 MB tumors. (A) Frequency distribution of cytogenetic aberrations (haploinsufficiency of 17p, i17q, c-Myc amplification) in (i) our local cohort of MB tumors (SHH n=9; group 3 MB n=10; group 4 MB n=16; Kanchan et al., GSE148390). (ii) Distinguishing features of high-risk tumors compared between our local cohort (Kanchan et al., GSE148390) and a larger dataset (Cavalli et al., GSE85217). (B) DNA methylation profile of local cohort of group 3 (n=6) and group 4 (n=11) MB tumors showing lack of perturbations to methylation in the promoter region of miR-212-3p compared to normal pediatric cerebellum (n=4); further demonstrable in vitro by a lack of expression restoration with de-methylation by 5-AzaC (5 µM) in HDMB03 cells. (C) Recapitulation of elevated HDAC and EZH2 expression complementing prior in silico data in a large MB meta-dataset (NC n=291, group 3 MB n=233; Weishaupt et al., GSE124814). (D) RT-PCR analysis in pan-HDAC-treated D425 cells (TSA, 100 nM; Belinostat, 1 µM; and Vorinostat, 1 µM) showing elevated expression of miR-212-3p. RNU6B set as an endogenous control. Western blotting analysis showed increased acetylated α-tubulin in pan-HDAC inhibitors treated D425 cells. β-actin served as an internal control. Results presented as mean ± SD from experiments done in triplicate and analyzed using Student’s t-test (B and D) or Mann-Whiteny U test (C); *p <0.05, **p <0.01, ***p <0.001. NC, normal cerebellum; dx, diagnosis. [file 40478_2021_1299_MOESM2_ESM.pdf]

Additional File 3: Fig. S2

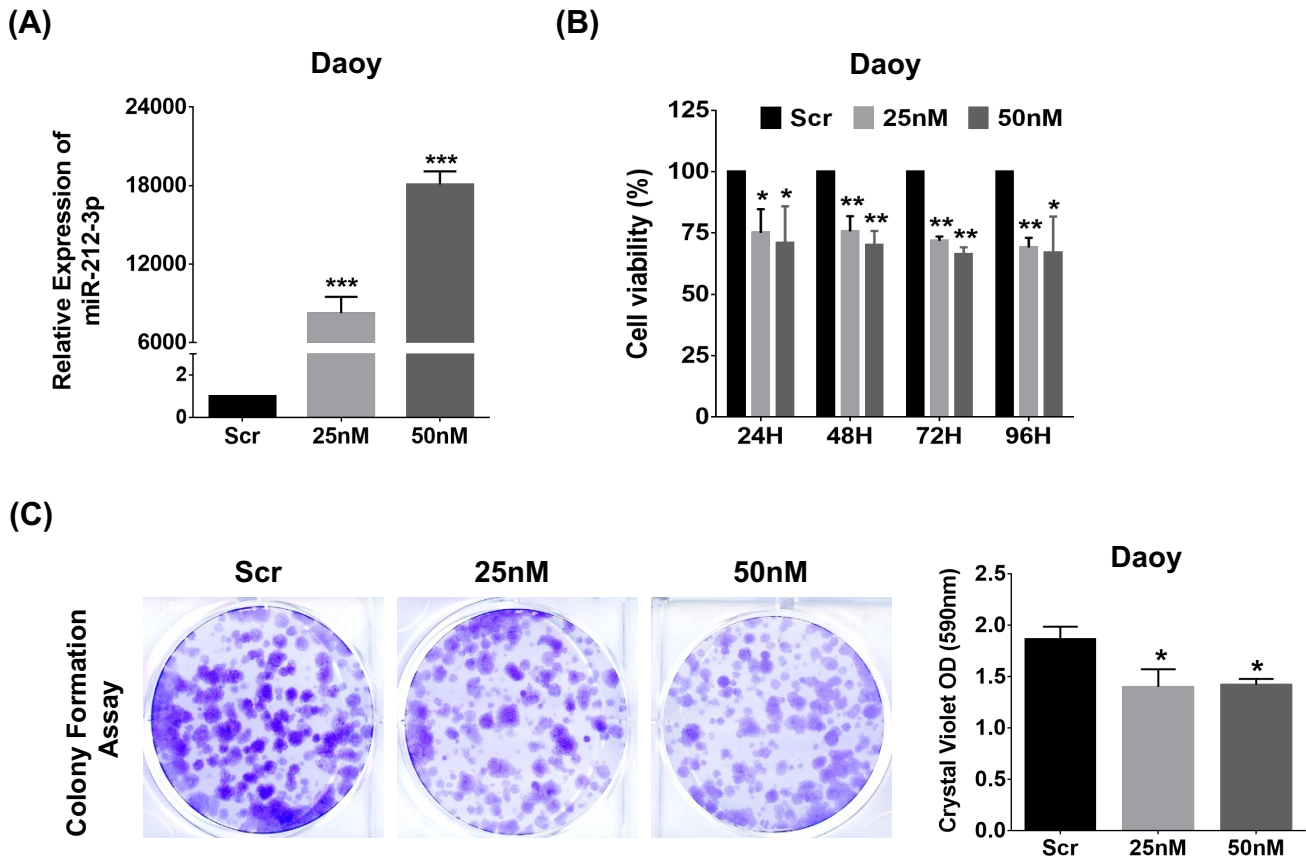

Supplement: Supplementary file 3 — Additional file 3: Fig. S2. Effect of miR-212-3p expression on SHH MB cancer cells. (A) RT-PCR analysis showing increased miR-212-3p expression via transient transfection in Daoy cells. Growth restrictive properties demonstrable by (B) cell proliferation (MTT) and (C) colony formation assays in miR-212-3p transfected Daoy cells. Data presented as mean ± SD from experiments done in triplicate and analyzed using Student’s t-test; *p <0.05, **p <0.01, ***p <0.001. [file 40478_2021_1299_MOESM3_ESM.pdf]

Additional File 4: Fig. S3

(A)

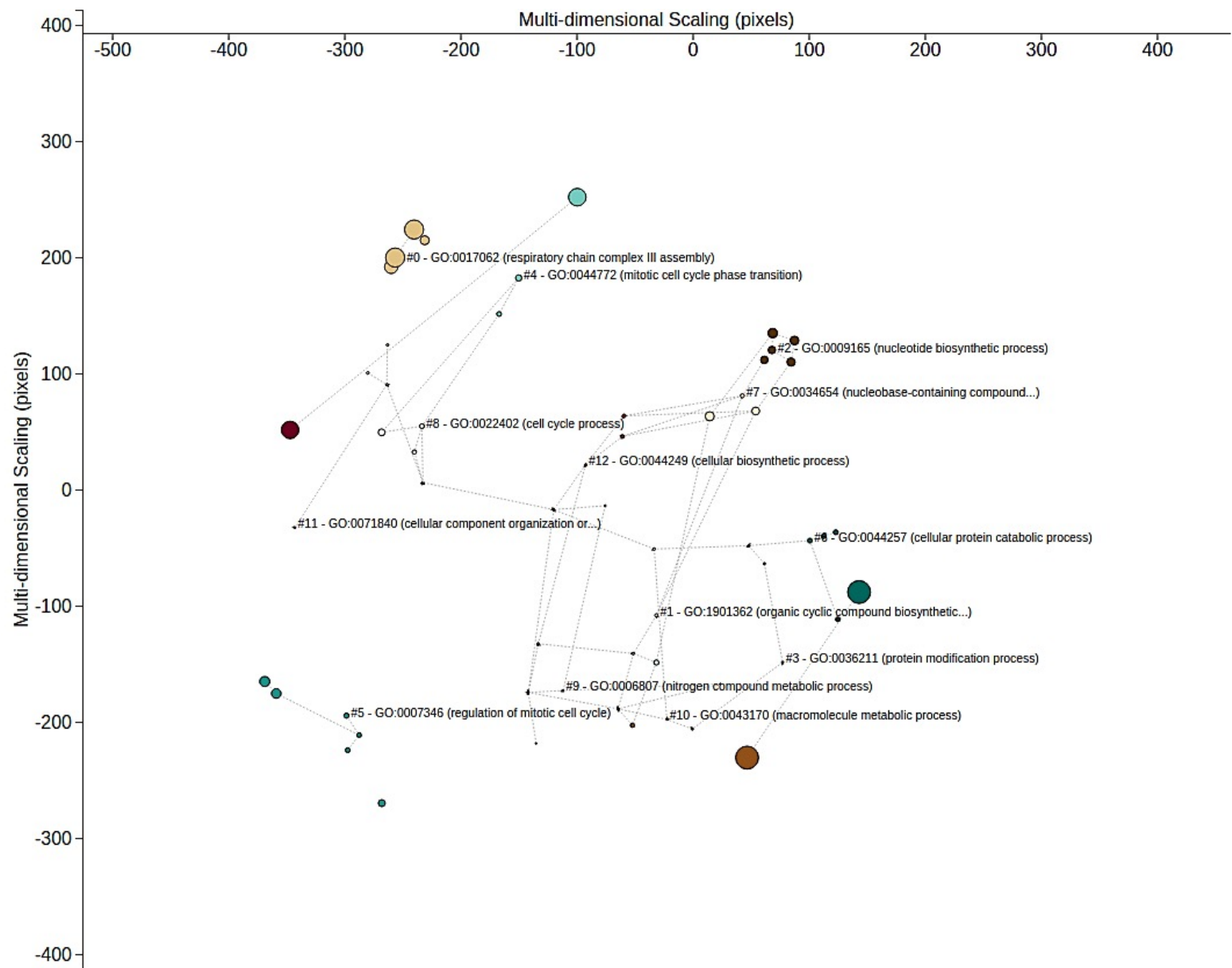

(B)

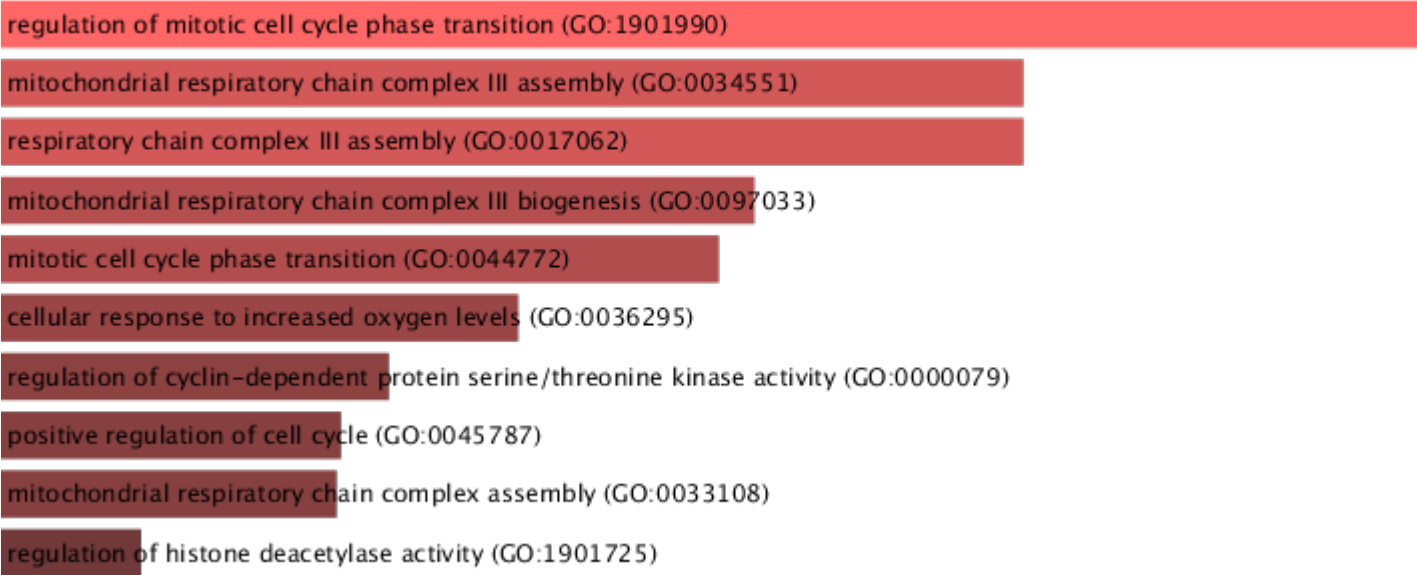

Supplement: Supplementary file 4 — Additional file 4: Fig. S3. Deregulated pathways associated with miR-212-3p silencing in group 3 MB. (A) Bubble plot (FunSet plot) representing 12 clusters of significantly enriched GO biological processes. FunSet employs hypergeometric test to perform the enrichments and additionally uses semantic similarity measure with Aggregate Information Content (AIC) index to cluster highly similar GO terms [17]. (B) Enriched biological pathways associated with miR-212-3p silencing identified using Enrichr (https://maayanlab.cloud/Enrichr/) [4] highlighting regulation of cell cycle phase transition as the most enriched pathway in miR-212-3p overexpressed HDMB03 cells [file 40478_2021_1299_MOESM4_ESM.pdf]

Additional File 6: Fig. S4

(A)

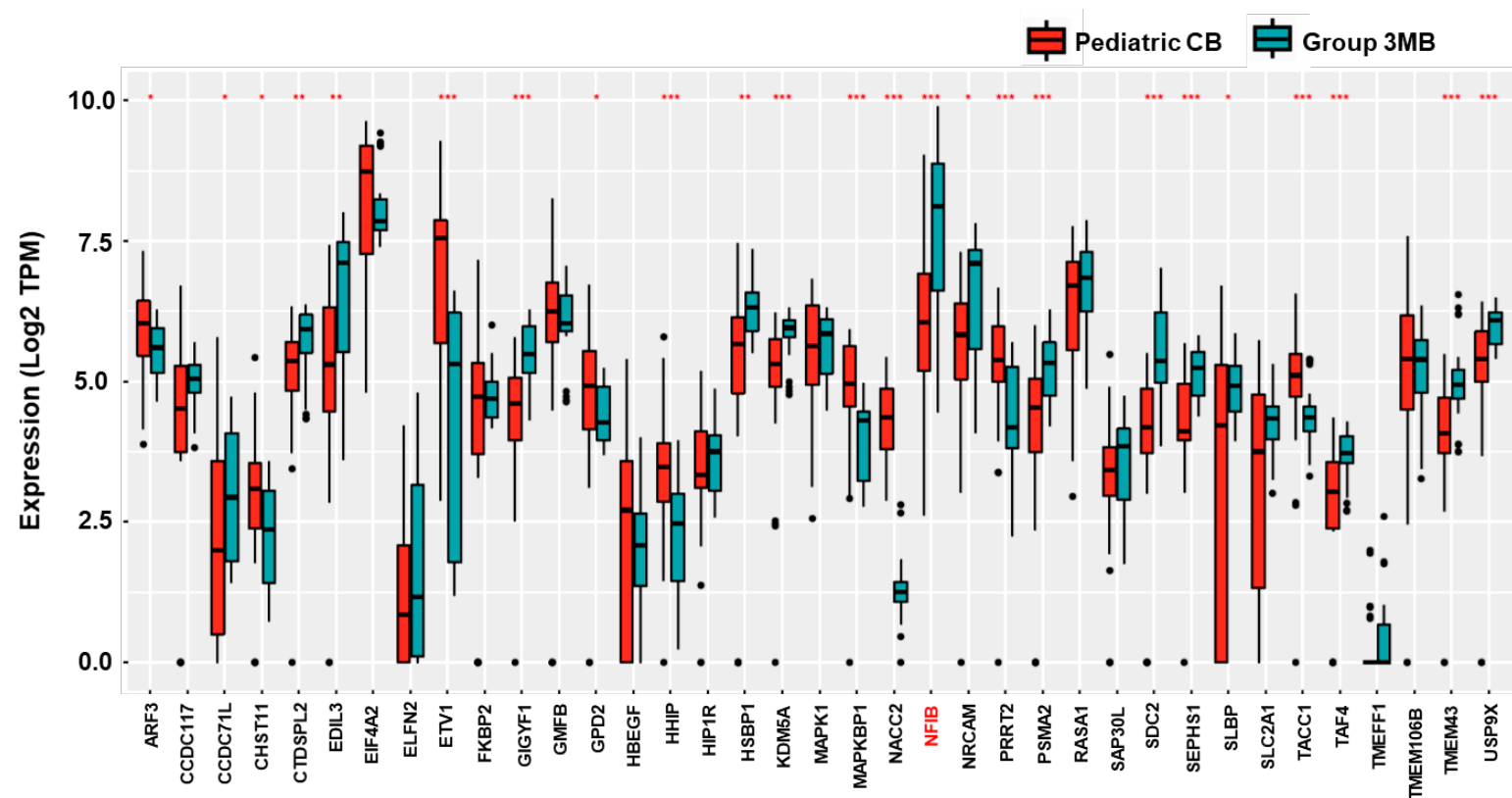

(B)

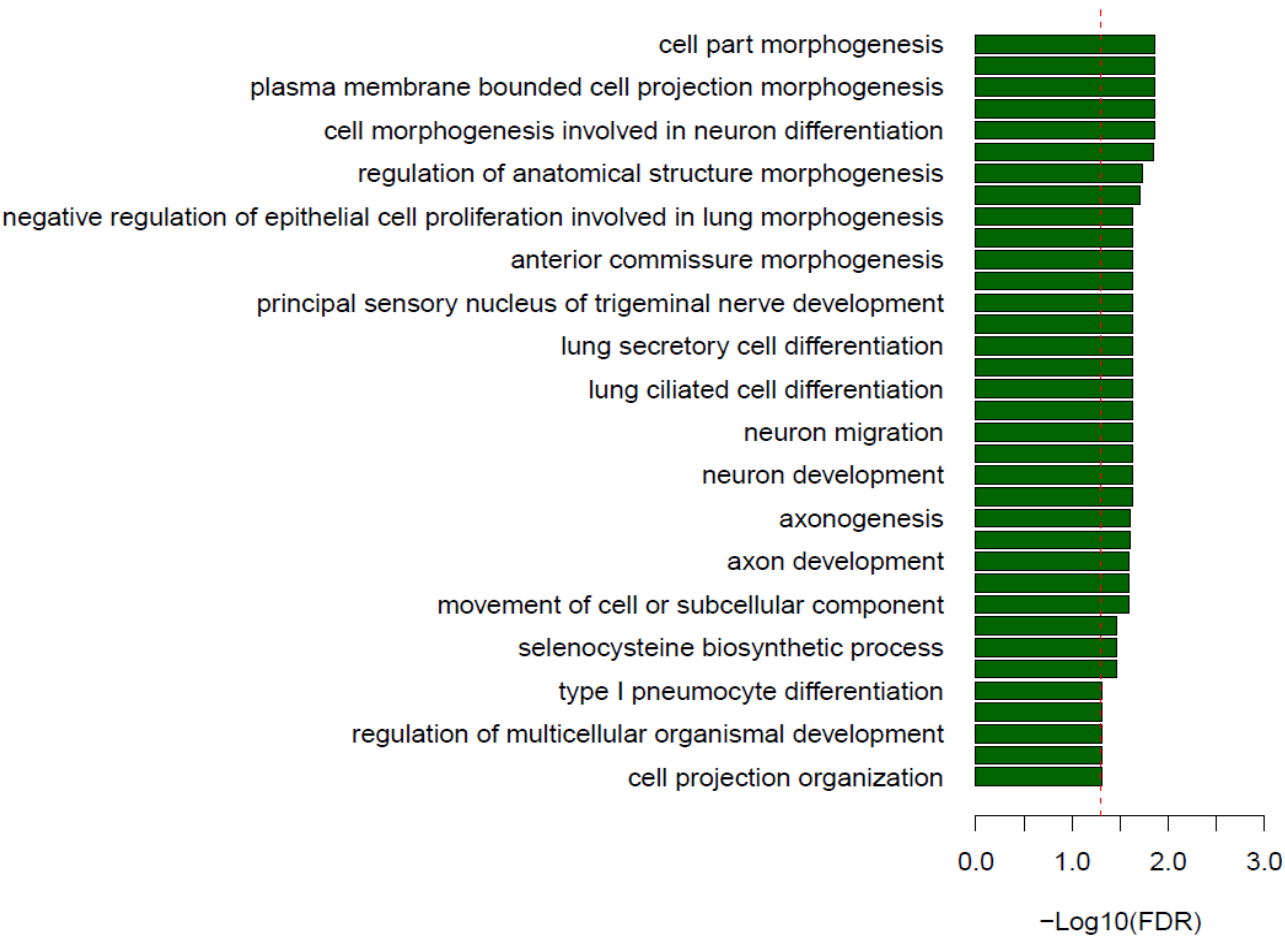

(C)

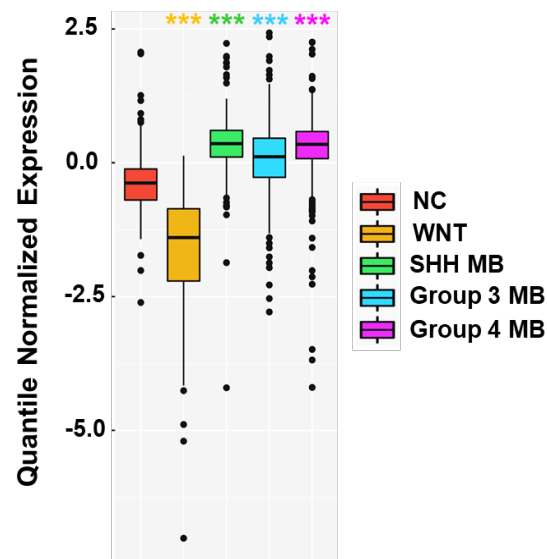

Supplement: Supplementary file 6 — Additional file 6: Fig. S4. Putative oncogenic targets of miR-212-3p and their associated pathways in group 3 MB tumors. (A) Expression analysis by RNA Sequencing of isolated miR-212-3p targets (37 genes) in local cohort of group 3 MB tumors (pediatric CB n=10; group 3 MB n=7; Kanchan et al., GSE148390). Those observed to be significantly elevated in group 3 tumors were the following 14 genes: CCDC71L, CTDSPL2, EDIL3, GIGYF1, HSBP1, KDM5A, NFIB, NRCAM, PSMA2, SDC2, SEPHS1, TAF4, TMEM43, and USP9X. (B) Enriched cellular/biological pathways associated with these targets identified using FunSet tool [17]. (C) Expression of NFIB across MB subgroups in a large MB meta-dataset (NC n=291; WNT n=118; SHH MB n=405; group 3 MB n=233; group 4 MB, n=530; Weishaupt et al., GSE124814). Box plots (A) represent gene expression (Log2 Transcripts Per Million) and were analyzed using Mann-Whiteny U test; *p <0.05, **p <0.01, ***p <0.001. Pathways represented as an inverted bar graph based on the -log10 FDR values with threshold p <0.05 (red dashed line). CB, cerebellum; NC, normal cerebellum. [file 40478_2021_1299_MOESM6_ESM.pdf]
