# Supplementary material for: MiR-212-3p functions as a tumor suppressor gene in group 3 medulloblastoma via targeting nuclear factor I/B (NFIB)
Source: Acta Neuropathol Commun. 2021 Dec 18;9:195. doi: 10.1186/s40478-021-01299-z (PMC8684142; doi:10.1186/s40478-021-01299-z)
Supplement: Supplementary file 5 — Additional file 5: Table S1. Genes (and associated pathways) enriched by miR-212-3p expression restoration in HDMB03 cells. Detailed gene list for selected clusters of significantly enriched GO biological processes represented in Additional file 4: Fig. S3A. Presented clusters were selected based on -log10 FDR values with threshold p <0.05. [file 40478_2021_1299_MOESM5_ESM.pdf]

Additional File 5: Table S1

Cluster	GO Name	FDR	Enrich. Score	Genes
4	response to increased oxygen levels	0.0394	10.4433	CCDC115 CAV1 ATP6AP1 ATP6V1A
4	mitotic cell cycle phase transition	0.0497	2.3319	MAX RPA1 AURKA CDC25B TUBB CCNB1 POLA2 CDKN2C HAUS2 OFD1 SKP2 CEP164 PRKAR2B BCAT1 PPP1R12B CUL4A ORC5 CEP63 CETN2
4	mitotic cell cycle process	0.0268	1.9073	MAX RPA1 NSL1 AURKA CDC25B TGFB1 TUBB CCNB1 BUB1B DCLRE1B MDC1 POLA2 PSRC1 CDKN2C RASA1 WRAP73 CENPE NCAPG HAUS2 OFD1 BLM SKP2 CEP164 INTS3 PRKAR2B SMC1A BCAT1 PPP1R12B BRSK1 CUL4A ORC5 CEP63 CETN2 NABP2 PTPA
5	regulation of cyclin-dependent protein serine/threonine kinase activity	0.0223	3.9001	CCNB1 PSRC1 CDKN2C PDGFB BLM CCNG2 TFAP4 CDK5RAP1 GTPBP4 MAPRE3 MEN1
5	regulation of cyclin-dependent protein kinase activity	0.0261	3.6734	CCNB1 PSRC1 CDKN2C PDGFB BLM CCNG2 TFAP4 CDK5RAP1 GTPBP4 MAPRE3 MEN1
5	regulation of G2/M transition of mitotic cell cycle	0.0458	2.5815	AURKA CDC25B TUBB CCNB1 PSMA2 HAUS2 HMMR OFD1 BLM CEP164 PRKAR2B USP47 BRSK1 CEP63 CETN2 PSMF1
5	regulation of mitotic cell cycle	0.0103	1.9859	AURKA CDC25B TGFB1 TUBB CCNB1 BUB1B DCLRE1B MDC1 XRCC3 PSRC1 CDKN2C PDXP IL1B PSMA2 PDGFB ANAPC15 CENPE PTTG1 HAUS2 HMMR OFD1 BLM LRP5 CEP164 CCNG2 INTS3 TFAP4 PRKAR2B SMC1A USP47 BRSK1 PPP1R9B NACC2 CEP63 CETN2 NABP2 CDC26 PSMF1 AURKAIP1
5	negative regulation of cell cycle	0.0292	1.9146	MAX AURKA TGFB1 TIMELESS CCNB1 BUB1B DCLRE1B MDC1 CAB39 MLF1 CDKN2C PSMA2 STK11 PTTG1 DHCR24 BLM INTS3 ING4 TFAP4 CDK5RAP1 SMC1A USP47 BRSK1 PPP1R9B CUL4A MEN1 NACC2 PCGF2 CEP63 NABP2 PSMF1 RHNO1 NUDT6 AURKAIP1
5	regulation of cell cycle	0.0012	1.8344	MAX AURKA CDC25B TGFB1 USP19 TIMELESS TUBB CCNB1 BUB1B DCLRE1B MDC1 FIGNL1 XRCC3 CAB39 PSRC1 MLF1 CDKN2C STAT3 PDXP IL1B PSMA2 PDGFB STK11 CDC42 LRP6 ANAPC15 CENPE PTTG1 HAUS2 HMMR DHCR24 OFD1 BLM LRP5 SKP2 CEP164 CCNG2 INTS3 ING4 TFAP4 PRKAR2B CDK5RAP1 G
8	cell cycle phase transition	0.0227	2.4922	MAX RPA1 AURKA CDC25B TIMELESS TUBB CCNB1 POLA2 SLBP CDKN2C HAUS2 OFD1 SKP2 CEP164 PRKAR2B BCAT1 PPP1R12B CUL4A ORC5 CEP63 CETN2
8	cell cycle process	0.0020	1.8962	MAX RPA1 NSL1 AURKA CDC25B TGFB1 TIMELESS TUBB CCNB1 BUB1B DCLRE1B CENPW MDC1 FIGNL1 POLA2 MND1 CAB39 PSRC1 SLBP MLF1 CDKN2C RASA1 STK11 WRAP73 CENPE NCAPG PTTG1 ANXA11 HAUS2 DHCR24 OFD1 BLM ASPM ARAP1 KNL1 SKP2 CEP164 INTS3 ING4 PRKAR2B MAPRE3 SMC1A BCAT
8	cell cycle	0.0073	1.7712	MAX RPA1 NSL1 AURKA CDC25B TGFB1 TIMELESS TUBB CCNB1 BUB1B DCLRE1B CENPW MDC1 FIGNL1 POLA2 MND1 CAB39 PSRC1 SLBP MLF1 CDKN2C RASA1 STK11 WRAP73 CENPE NCAPG PTTG1 ANXA11 HAUS2 DHCR24 OFD1 BLM ASPM ARAP1 KNL1 SKP2 CEP164 CCNG2 INTS3 ING4 PRKAR2B MAPRE3 SMC1
8	organelle organization	0.0012	1.4390	GAPDH RPA1 NSL1 AURKA TUBB SHROOM3 GSTK1 AP2M1 PARP14 RAB34 CCNB1 BUB1B DCLRE1B CENPW FIGNL1 XRCC3 POLA2 MND1 PSRC1 NRP1 PDXP CCDC115 HDAC5 SOD2 RAC3 CDC42 ARHGAP35 NUP93 NDUFAF3 TMEM138 WRAP73 CENPE NCAPG PTTG1 CAV1 LAPTM4B HAUS2 HUWE1 ZNF335 OFD1 ARL6 A
8	cellular component organization	0.0003	1.3398	GAPDH RPA1 RABEP1 NSL1 COL5A1 AURKA ITGA3 ITGB4 COL6A2 TGFB1 TUBB TGFBI SHROOM3 GSTK1 AP2M1 SLC2A1 PARP14 RAB34 TK1 CCNB1 BUB1B DCLRE1B CENPW FIGNL1 XRCC3 POLA2 MND1 DNASE2 PSRC1 SLITRK4 ZC4H2 NRP1 COL18A1 PDXP CCDC115 ANXA6 HBEGF ITGAV HDAC5 SMAD4 PDGFB
8	cellular process	0.0314	1.0651	GAPDH G6PC3 MAX RPA1 UMPS CTSB HSBP1 NUP88 RABEP1 NSL1 CPM COL5A1 IL11 PLAU AURKA CDC25B ITGA3 ITGB4 COL6A2 TGFB1 USP19 TIMELESS TUBB TGFBI SHROOM3 GSTK1 AP2M1 CHST11 SLC26A6 SLC2A1 PARP14 RAB34 TK1 GMPS SLC30A9 GCOM1 LGALS1 MOGS CCNB1 BUB1B DCLRE1B CENPW
