# Supplementary material for: MiR-212-3p functions as a tumor suppressor gene in group 3 medulloblastoma via targeting nuclear factor I/B (NFIB)
Source: Acta Neuropathol Commun. 2021 Dec 18;9:195. doi: 10.1186/s40478-021-01299-z (PMC8684142; doi:10.1186/s40478-021-01299-z)
Supplement: Supplementary file 7 — Additional file 7: Table S2. Isolating a strong oncogenic target of miR-212-3p. Further analysis of putative miR-212-3p targets by examining conserved binding sites, confirming elevated expression in 2 independent MB cohorts, and examining effect of high expression on survival. In the table, ‘x’ denotes inclusion criteria, and ‘-’ denotes no expression data found in dataset. [file 40478_2021_1299_MOESM7_ESM.pdf]

Additional File 7: Table S2

MiR-212-3p Targets	3'-UTR Binding Sites, # (Target Scan)	Elevated Expression in Group 3 MB		KM Survival Data (GSE85217)	
		GSE148390 (n=10)	GSE124814 (n=233)	High Expressing patients (%)	High Expression = Poor Survival
CCDC71L	1	x	x	-	
CTDSPL2	1	x	x	97.2%	
EDIL3	1	x	x	62.09%	x
GIGYF1	1	x		82.67%	
HSBP1	1	x	x	73.69%	
KDM5A	2	x	-	1.30%	x
NFIB	2	x	x	88.56%	x
NRCAM	1	x		19.93%	
PSMA2	1	x	x	1.30%	x
SDC2	1	x	x	65.68%	x
SEPHS1	1	x	x	3.75%	x
TAF4	2	x	x	50.16%	x
TMEM43	1	x	x	89.37%	
USP9X	1	x		23.36%	x
